# Temporal relationship between severe mental illness and neurological conditions in a UK primary care cohort

**DOI:** 10.1136/bmjment-2025-301923

**Published:** 2025-11-04

**Authors:** Ella Burchill, Jonathan P Rogers, David P J Osborn, Glyn Lewis, Anthony S David, Joseph F Hayes, Naomi Launders

**Affiliations:** 1 Division of Psychiatry, University College London, London, UK; 2 Department of Neuropsychiatry, National Hospital for Neurology and Neurosurgery, London, UK; 3 North London NHS Foundation Trust, London, UK; 4 Institute of Mental Health, Division of Psychiatry, University College London Faculty of Brain Sciences, London, UK

**Keywords:** Schizophrenia, Schizophrenia Spectrum and Other Psychotic Disorders, Neurocognitive Disorders, Bipolar and Related Disorders, Affective Disorders, Psychotic

## Abstract

**Background:**

A higher prevalence of neurological conditions has been found in schizophrenia, bipolar disorder and other psychotic illnesses compared to the general population. We aimed to understand the cumulative prevalence of 15 neurological conditions in people with severe mental illness (SMI) from 5 years before to 5 years after their SMI diagnosis.

**Methods:**

We identified patients with SMI, aged 18–100 years from 1 Jan 2000 to 31 Dec 2018, from the UK Clinical Practice Research Datalink. Each SMI patient was matched 1:4 to individuals without SMI. The cumulative prevalence of 15 neurological conditions was recorded at 5, 3 and 1 years prior to SMI diagnosis; at SMI diagnosis; and 1, 3 and 5 years after SMI diagnosis. Prevalences were compared with logistic regression.

**Results:**

We identified 68 789 patients with SMI and 274 827 comparators. Of 15 neurological conditions, 13 (multiple sclerosis, cerebrovascular disease, dementia, ataxic disorders, epilepsy, Parkinson’s disease, other parkinsonism, paralysis, other movement disorders, cerebrospinal fluid disorders, cerebral palsy, disorders of nerve root, plexus or peripheral nerves and autonomic disorders) were more prevalent in SMI compared with comparators at the time of SMI diagnosis. Dementia (OR: 4.22; 95% CI 3.88 to 4.58), epilepsy (OR: 3.01; 95% CI 2.83 to 3.19) and Parkinson’s disease (OR: 3.97; 95% CI 3.45 to 4.57) were particularly elevated at 5 years post-SMI diagnosis.

**Conclusions:**

Many neurological conditions have higher prevalence in the SMI cohort compared with those without SMI. The different prevalence patterns observed in our study highlight the need to establish the causal pathways between specific SMI and neurological disease diagnoses.

**WHAT IS ALREADY KNOWN ON THIS TOPIC**

- Neurological conditions are overrepresented in people with severe mental illness, but how this differs between neurological conditions and the temporal relationships is not clear.

**WHAT THIS STUDY ADDS**

- Examining 15 categories of neurological conditions over seven timepoints relative to diagnosis of severe mental illness, we found 13 conditions were overrepresented. Dementia, epilepsy and Parkinson’s disease had the strongest relationship to severe mental illness.
- Different neurological conditions tend to appear at different times relative to a diagnosis of severe mental illness, but there was often an increase in incidence around the time of the psychiatric diagnosis.

**HOW THIS STUDY MIGHT AFFECT RESEARCH, PRACTICE OR POLICY**

- Clinicians must be particularly vigilant for dementia, epilepsy and Parkinson’s disease in the years around a diagnosis of severe mental illness.
- Future research should address the causal relationships between neurological conditions and severe mental illness.

## Introduction

 There is a well-established relationship between schizophrenia, bipolar affective disorder and other psychotic disorders, which we term severe mental illness (SMI), on the one hand, and physical morbidity on the other.^1^ This spans a wide range of physical conditions, including cardiovascular, endocrine, pulmonary and gastrointestinal disorders.^2^ There has been considerable focus on cardiometabolic disease in SMI, with other comorbidities receiving less research attention.^3^,^5^ In particular, the relationship between psychiatric and neurological conditions is not fully understood.^6 7^ Neurological disorders are the most common cause of complex multimorbidity in individuals with SMI, consequently leading to disability.^8^ Previous work by Launders and colleagues found the prevalence of neurological conditions to be much higher before, at the time of and after SMI diagnosis, compared with those without SMI.^9^

The traditional categorical separation between SMI and some neurological conditions may result in underestimation of the true burden of mental illness and misdiagnosis between groups. Many common neurological conditions, such as Parkinson’s disease, multiple sclerosis and epilepsy, are increasingly considered ‘neuropsychiatric’ in nature, recognising the co-existing neurological, psychiatric and cognitive symptom burden. The majority of prevalence studies thus far have been cross-sectional.^10^,^12^ This limits our understanding of the temporal relationship between neurological and psychiatric disorder presentation.

Notwithstanding these limitations, it is clear that there are some specific temporal relationships between psychotic symptoms and certain neurological disorders. For example, in Parkinson’s disease, psychotic symptoms are common and tend to become more prevalent and more severe over time as the disease progresses.^12^ In epilepsy, there are several distinct psychotic symptoms characterised by different temporal relationships to the seizures, specifically ictal psychosis (during a seizure), postictal psychosis (in the days or weeks after a seizure) and interictal psychosis (with no clear relationship to seizures).^13^ In addition, psychosis can emerge due to treatment with or withdrawal from antiseizure medications.^14 15^ In the spinocerebellar ataxias (SCA), there have been case reports and series of often chronic and sometimes progressive psychosis in SCA-2, SCA-3, SCA-7, SCA-10 and SCA-17.^16^,^21^ These findings, however, leave many important lacunae in our current understanding. Crucially, it is generally not clear to what extent patients with psychotic symptoms would meet formal diagnostic criteria and would qualify as having SMI. Moreover, it is not known whether all these findings are replicable in large datasets, as they often rely on small, single-centre studies. Finally, it is not certain to what extent findings in one neurological disorder are generalisable to others or whether each neurological condition has a different pattern in its temporal relationship to SMI.

A better understanding of the temporal association between individual neurological conditions and SMI is vital to understand causality in what may be a bidirectional relationship. The comorbidity may be due to a psychiatric prodrome to a neurological disorder, a neurological prodrome to a psychiatric disorder, a causal relationship in either direction or due to shared underlying genetic or pathological mechanisms. Investigating the distinct time points of both neurological and psychiatric diagnoses could promote earlier diagnosis and management, with the goal of timely prevention and intervention to reduce excess disability and mortality. We aimed to understand the cumulative prevalence of 15 neurological conditions, diagnosed in people with SMI from 5 years before to 5 years after their first recorded SMI diagnosis. We hypothesised that the individual neurological conditions would have differential temporal relationships relative to SMI diagnosis in comparison to diagnoses in people without SMI. Given that certain neurological conditions are comparatively rare, we chose to use large-scale primary care records. Secondary care records may be more likely to miss chronic, stable neurological conditions, such as cerebral palsy.

## Methods

### Study design and participants

We identified individuals with SMI from the primary care database Clinical Practice Research Datalink (CPRD) (Aurum and Gold) and conducted analyses at seven time points relative to SMI diagnosis. The CPRD is broadly representative of the UK population in terms of age, sex, ethnicity and region, comprising data from approximately 24% of the UK population. As one of the largest databases of longitudinal electronic healthcare records globally, it has been previously validated for epidemiological research for a wide range of clinical presentations, including among diagnoses generally made in secondary care, which are transferred to the patient record by general practitioners.

Our cohort of interest received a first diagnosis of schizophrenia, bipolar disorder or other psychosis, between 1 Jan 2000 and 31 Dec 2018 (considered the index date), and were aged 18–100. In individuals with multiple SMI diagnoses, the latest diagnostic category was used, as clinicians had more of a longitudinal trajectory on which to base diagnostic decisions; however, the date from the first SMI diagnosis was still used as the index date. Each individual with SMI was matched by CPRD with up to four individuals without SMI from the same primary care practice, by sex, age within 5 years and year of practice registration. Up to four controls were selected, as gains in statistical efficiency become slight after this point and increasing the number might bias the results^22^ because certain cases would potentially have many more eligible controls than others. An index date for the matched individuals was taken as the index date of the SMI diagnosis in the individuals with whom they were matched. Patients who changed to a different general practice during the study were censored at this point. The cohort has been described in detail previously.^9^

### SMI and neurological diseases

We identified three subtypes of SMI (schizophrenia, bipolar disorder or other non-affective, non-organic psychotic illness) from medical codes recorded in primary care records (codes available in online supplemental table 4). The first date of any SMI diagnosis was used to represent date of diagnosis during the study period.

Neurological conditions were grouped into disease clusters, which were used to develop ICD-10 code lists for extraction from the primary care records (codes available in online supplemental table 4). The resulting 15 neurological clusters were: multiple sclerosis (or other white matter disorders), cerebrovascular disease, dementia (inclusive of Alzheimer’s, Lewy body, vascular, human prion disorders), ataxic disorders, epilepsy (or seizures), Parkinson’s disease, parkinsonism other (secondary/drug induced), paralysis, movement disorders other (choreiform, movement, dystonic), structural developmental anomalies or disorders of cerebrospinal fluid (CSF) pressure or flow, cerebral palsy, spinal cord disorders, disorders of nerve root, plexus or peripheral nerves, motor neuron diseases or related disorders and disorders of autonomic nervous system. We excluded acute neurological presentations, including delirium and encephalopathy,^23^ as we wished to focus on stable neurological diagnoses and also did not want to risk contamination of the ‘other psychosis’ SMI subcohort. We excluded any drug poisoning medical codes for similar reasons. If both hydrocephalus and spina bifida were coded, the diagnostic category of hydrocephalus was used in preference. Due to insufficient numbers, we combined the ICD-10 categories of CSF fluid/pressure flow disorders and structural developmental anomalies of the nervous system into one umbrella category.

Although diagnoses of SMIs and neurological conditions are often made in secondary care, these diagnoses are then imported into primary care records and there is evidence for good validity of these diagnoses in CPRD.^24^

### Confounders

All confounders were defined, a priori, using a directed acyclic graph (see online supplemental figure 1. Our primary model adjusted for sex, age at SMI diagnosis, ethnicity, region and year of SMI diagnosis. We classified ethnicity using the UK 2011 Census ethnic groups: Asian, Black, Mixed, White or Other.

In a further model, we additionally adjusted for body mass index (BMI), smoking, alcohol misuse and substance misuse. This model therefore examines the relationship between neurological conditions and SMI, independent of these covariates: in some neurological conditions, these may be confounders; in others, they may be on the causal pathway. BMI was categorised as obese (BMI ≥30), overweight (BMI 25–29.9), within normal range (BMI 18.5–24.9) or underweight (BMI <18.5). Smoking status was recorded as current-smoker, ex-smoker and never-smoked. Alcohol and substance misuse were defined binarily using the code lists for the Elixhauser comorbidity index. BMI, alcohol and substance misuse were captured at 5 years before SMI diagnosis, and if this was not available, preference was given to data recorded prior to SMI diagnosis.

### Statistical analysis

We calculated the cumulative prevalence of the 15 neurological conditions at 5 years, 3 years and 1 year before SMI diagnosis; at the point of diagnosis (index); and 1 year, 3 years and 5 years after diagnosis. In each case, the denominator was the number of patients alive and available for follow-up at that timepoint. We used logistic regression to compare the odds of each individual neurological condition in those with and without SMI at each timepoint, further stratifying the results by SMI subtype. Sandwich standard errors were applied to account for potential clustering by primary care practice. All analysis was conducted in R V.4.2.1 and RStudio 2022.07.2. The *R* code for the study is available at https://github.com/NaomiLaunders/NeuroInSMI.

ORs were calculated for unadjusted, primary (adjusting for sex, age at SMI diagnosis, ethnicity, region and year of SMI diagnosis) and additionally adjusted models (inclusive of all the above and BMI, smoking, alcohol misuse and substance misuse).

### Missing data

In cases of missing ethnicity or BMI data, multiple imputation was conducted using the *mice* package V.3.16.0, pooling five imputations together in accordance with Rubin’s rule. In cases of missing data for smoking status, this was imputed as never-smoker, consistent with other studies suggesting GPs less commonly record values they perceive to be normal. For all other clinical variables, absence of a record was assumed to represent absence of the condition.

## Results

We identified 68 789 patients with SMI and 274 827 matched comparator patients after excluding 2066 patients from the SMI group and 8571 from the comparator group. Full exclusion reasons and numbers are presented in online supplemental table 2. In the SMI cohort, the median age at SMI diagnosis was 41 years, and 49% were female (33 783 SMI patients). During the 5 years after SMI diagnosis, 4524 (6.58%) of those with SMI died, compared with 11 206 (4.08%) of the non-SMI group. Other patient characteristics are shown in table 1.

**Table 1:** Patient characteristics by SMI diagnosis and subtype

	SMI cohort	Schizophrenia	Bipolar	Other psychoses	Matched population
N	68 789	15 034	24 423	29 332	274 827
Age at diagnosis, years (median (IQR))*	40.92 (29.47, 56.05)	38.15 (27.90, 51.37)	41.78 (31.17, 54.70)	41.74 (29.00, 60.95)	40.90 (29.46, 56.00)
Female sex (%) **†**	33 738 (49.05)	5343 (35.54)	14 450 (59.17)	13 945 (47.54)	134 740 (49.03)
Ethnicity (%)					
Asian	3134 (4.56)	1030 (6.85)	749 (3.07)	1355 (4.62)	13 536 (4.93)
Black	3389 (4.93)	1359 (9.04)	486 (1.99)	1544 (5.26)	8566 (3.12)
Mixed	913 (1.33)	277 (1.84)	243 (0.99)	393 (1.34)	2271 (0.83)
Other	1486 (2.16)	331 (2.20)	502 (2.06)	653 (2.23)	5888 (2.14)
White	35 228 (51.21)	6594 (43.86)	14 284 (58.49)	14 350 (48.92)	125 518 (45.67)
Missing	24 639 (35.82)	5443 (36.20)	8159 (33.41)	11 037 (37.63)	119 048 (43.32)
Body mass index category (**%**)					
Underweight (<18.5)	2248 (3.27)	500 (3.33)	630 (2.58)	1118 (3.81)	6211 (2.26)
Normal (18.5-<25)	22 648 (32.92)	4262 (28.35)	8321 (34.07)	10 065 (34.31)	89 980 (32.74)
Overweight (25-<30)	14 595 (21.22)	2761 (18.37)	5793 (23.72)	6041 (20.60)	61 852 (22.51)
Obese (>30)	9981 (14.51)	2010 (13.37)	4137 (16.94)	3834 (13.07)	35 883 (13.06)
Missing	19 317 (28.08)	5501 (36.59)	5542 (22.69)	8274 (28.21)	80 901 (29.44)
Smoking status (%) ‡					
Current smokers	30 024 (43.65)	6821 (45.37)	10 706 (43.84)	12 497 (42.61)	83 057 (30.22)
Ex-smokers	8610 (12.52)	1336 (8.89)	3503 (14.34)	3771 (12.86)	38 978 (14.18)
Never smokers	30 155 (43.84)	6877 (45.74)	10 214 (41.82)	13 064 (44.54)	152 792 (55.60)
Substance misuse (%) ‡	5642 (8.20)	1280 (8.51)	1455 (5.96)	2907 (9.91)	3968 (1.44)
Alcohol misuse (**%**) ‡	6093 (8.86)	1096 (7.29)	2157 (8.83)	2840 (9.68)	6232 (2.27)
Died during study (%)‡	4524 (6.58)	851 (5.66)	1143 (4.68)	2530 (8.63)	11 206 (4.08)
Age at death (median (IQR))	75.71 (59.46, 86.04)	68.15 (52.50, 81.15)	72.87 (59.63, 82.87)	80.91 (62.95, 88.98)	82.67 (72.24, 89.34)
Follow-up after index date, years (median (IQR))	4.60 (2.39, 8.71)	5.21 (2.64, 9.93)	5.06 (2.63, 9.12)	4.01 (2.15, 7.68)	4.58 (2.10, 8.93)
Baseline follow-up, years (median (IQR))	5.95 (1.04, 16.19)	3.40 (0.27, 12.65)	6.04 (1.15, 15.68)	7.32 (1.62, 18.10)	8.81 (2.82, 18.85)

* Eligibility criteria for the study required participants to be aged 18 to 100 at diagnosis, so there were no missing values for age.

† Clinical Practice Research Datalink do not provide data for participants without a recorded sex, so there were no missing values.

‡ For clinical variables, the absence of a clinical code was taken to denote the absence of the condition of interest.

SMI, severe mental illness.

**Figure 1:**
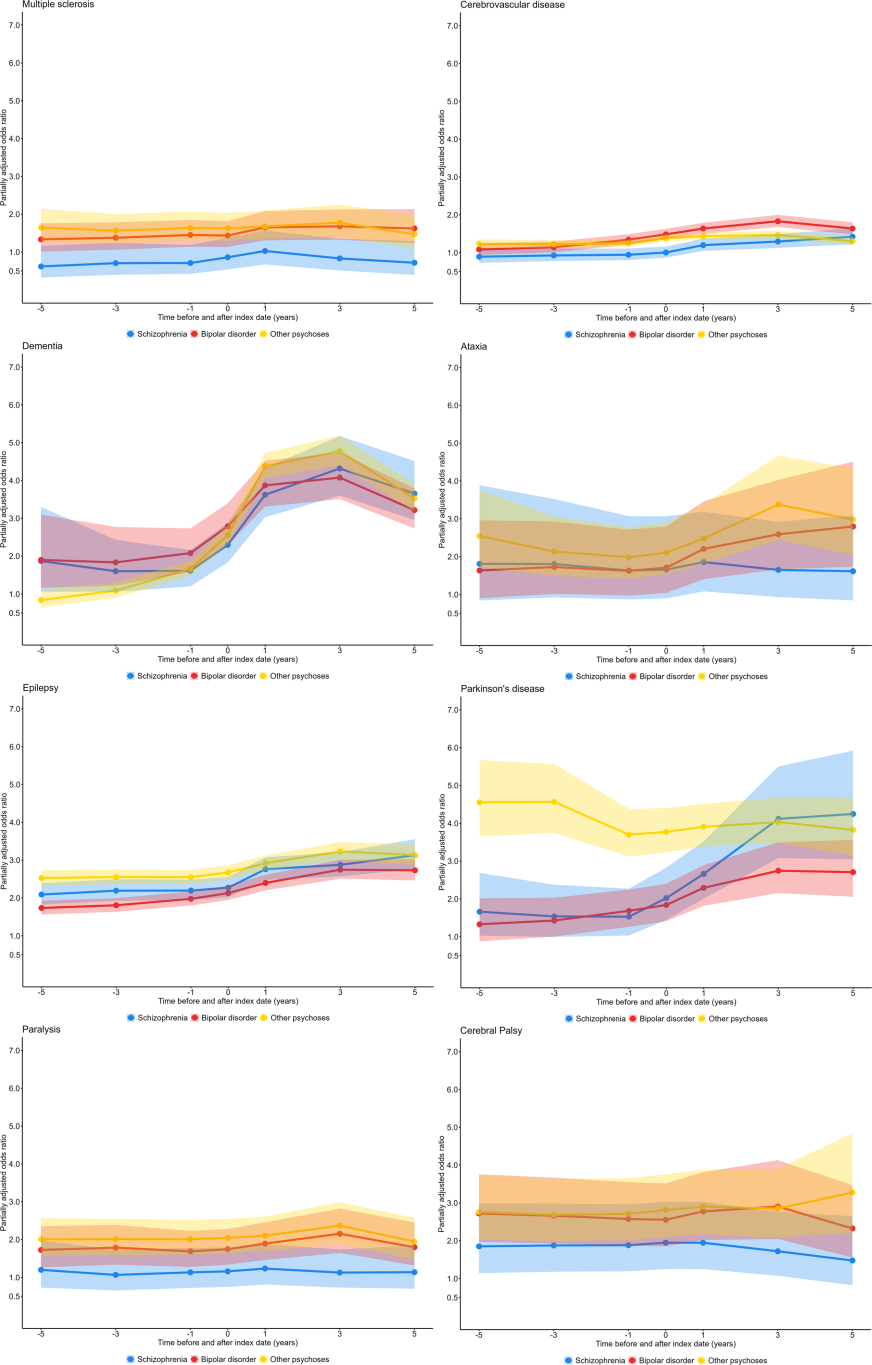
Odds ratios for selected neurological conditions, adjusted for sex, age at SMI diagnosis, ethnicity, region and year of SMI diagnosis at all time points for people with schizophrenia, bipolar disorder and other psychoses compared with matched population without SMI. SMI, severe mental illness.

At the date of first receiving their SMI diagnosis in primary care, the five most commonly comorbid neurological conditions were: epilepsy (3.71%), cerebrovascular disease (3.02%), dementia (1.65%), Parkinson’s disease (0.68%) and multiple sclerosis (0.32%) (online supplemental table 3). Unadjusted ORs for the relative prevalence of neurological conditions in people with SMI compared with a comparator population are shown in online supplemental table 5. After adjusting for sex, age at SMI diagnosis, ethnicity, region and year of SMI diagnosis, people with SMI had elevated prevalence of 11 out of 15 neurological conditions at all time points (including those prior to diagnosis) and 13 out of 15 at the time of SMI diagnosis (table 2). Only motor neuron disease was never elevated in people with SMI compared with those without SMI. The most elevated ORs at the time of SMI diagnosis were for parkinsonism (other) (OR 8.30; 95% CI 5.60 to 12.28) and other movement disorders (OR 3.04; 95% CI 2.10 to 4.40), while dementia, Parkinson’s disease, other movement disorders, epilepsy and cerebral palsy all had ORs above two (table 2). Dementia and Parkinson’s disease had the greatest increase in ORs over the time period considered (figure 1, online supplemental figure 2), and were particularly elevated at 5 years post-SMI diagnosis (dementia OR: 4.22; 95% CI 3.88 to 4.58, Parkinson’s disease OR: 3.97; 95% CI 3.45 to 4.57, table 2).

**Table 2:** Relative prevalence of neurological conditions in people with SMI to the comparator population (ORs adjusted for sex, age at SMI diagnosis, ethnicity, region and year of SMI diagnosis)

Condition	Years before SMI diagnosis			Index date	Years after SMI diagnosis		
	-5	-3	-1		1	3	5
Multiple sclerosis	1.36 (1.13–1.62)	1.36 (1.15–1.61)	1.41 (1.21–1.65)	1.43 (1.23–1.66)	1.57 (1.35–1.82)	1.52 (1.30–1.79)	1.43 (1.19–1.72)
Cerebrovascular disease	1.14 (1.06–1.23)	1.16 (1.09–1.24)	1.22 (1.16–1.29)	1.35 (1.28–1.42)	1.45 (1.38–1.52)	1.59 (1.51–1.67)	1.63 (1.53–1.73)
Dementia	1.12 (0.88–1.42)	1.27 (1.07–1.51)	1.72 (1.55–1.91)	2.54 (2.34–2.76)	4.10 (3.84–4.37)	4.85 (4.53–5.19)	4.22 (3.88–4.58)
Ataxia	2.12 (1.58–2.85)	1.94 (1.48–2.56)	1.81 (1.40–2.34)	1.91 (1.50–2.43)	2.27 (1.79–2.89)	2.73 (2.12–3.50)	2.72 (2.08–3.55)
Epilepsy	2.15 (2.03–2.28)	2.21 (2.10–2.34)	2.27 (2.16–2.40)	2.40 (2.28–2.52)	2.70 (2.57–2.83)	2.87 (2.73–3.03)	3.01 (2.83–3.19)
Parkinson’s disease	3.09 (2.60–3.67)	3.10 (2.66–3.62)	2.81 (2.45–3.21)	2.96 (2.62–3.34)	3.25 (2.91–3.63)	3.75 (3.35–4.20)	3.97 (3.45–4.57)
Paralysis	1.78 (1.48–2.15)	1.77 (1.49–2.11)	1.75 (1.48–2.06)	1.79 (1.52–2.10)	1.88 (1.60–2.20)	1.99 (1.68–2.35)	1.76 (1.45–2.13)
Cerebral palsy	2.56 (2.09–3.12)	2.50 (2.05–3.04)	2.48 (2.04–3.02)	2.52 (2.08–3.05)	2.64 (2.17–3.20)	2.43 (1.97–2.99)	2.34 (1.83–3.00)
CSF disorders	1.80 (1.31–2.46)	1.75 (1.29–2.38)	1.81 (1.35–2.44)	1.96 (1.47–2.62)	2.21 (1.64–2.99)	2.26 (1.63–3.13)	2.34 (1.65–3.30)
Spinal cord disorders	1.30 (0.84–2.02)	1.37 (0.92–2.04)	1.44 (1.01–2.04)	1.59 (1.15–2.20)	1.70 (1.25–2.31)	1.70 (1.22–2.38)	1.45 (0.99–2.12)
Disorders of nerve root, nerve plexus and peripheral nervous system	1.38 (0.96–1.98)	1.26 (0.90–1.78)	1.38 (1.00–1.92)	1.51 (1.11–2.05)	1.70 (1.26–2.30)	1.96 (1.44–2.68)	2.03 (1.46–2.83)
Parkinsonism (other)	7.70 (3.68–16.13)	9.71 (5.31–17.78)	6.25 (3.97–9.85)	8.32 (5.64–12.29)	10.59 (7.63–14.70)	14.51 (10.16–20.72)	19.08 (12.65–28.78)
Movement disorders (other)	1.93 (1.20–3.12)	2.35 (1.53–3.62)	2.50 (1.68–3.71)	3.03 (2.10–4.39)	3.71 (2.59–5.33)	4.13 (2.78–6.14)	4.42 (2.94–6.63)
Motor neuron disease	0.72 (0.21–2.51)	0.53 (0.16–1.78)	0.72 (0.28–1.90)	0.72 (0.28–1.90)	1.59 (0.58–4.35)	1.50 (0.47–4.78)	1.79 (0.68–4.69)
Autonomic nervous system disorders	1.58 (0.68–3.66)	1.89 (0.87–4.09)	1.80 (0.84–3.87)	1.98 (0.94–4.16)	1.98 (0.94–4.16)	3.40 (1.51–7.66)	3.39 (1.32–8.70)

SMI, severe mental illness.

When stratified by SMI subtypes, only the prevalence of epilepsy and cerebral palsy was elevated in all three SMI subtypes at 5 years before SMI diagnosis when adjusting for sex, age at SMI diagnosis, ethnicity, region and year of SMI diagnosis (online supplemental table 6, figure 1, online supplemental figure 2). However, at the time of SMI diagnosis, the prevalence of dementia and Parkinson’s disease was also elevated in all three subtypes. The prevalence of multiple sclerosis, cerebrovascular disease, paralysis and CSF disorders was elevated at the time SMI diagnosis for patients with bipolar and other psychoses but similar to the comparator population for those with schizophrenia, while spinal fluid disorders were only elevated in those with bipolar disorder, and disorders of nerve root, nerve plexus and peripheral nervous system were not elevated at the time of SMI diagnosis for any SMI diagnosis. Despite being elevated for other subtypes of SMI, multiple sclerosis, paralysis, disorders of nerve root, nerve plexus and peripheral nervous system and spinal cord disorders were not elevated for patients with schizophrenia at any time points.

We did not stratify by specific SMI diagnosis for parkinsonism (other), other movement disorders, motor neuron disease and autonomic nervous system disorders as these conditions were reported in less than 100 people (<0.04%) without SMI at all time points.

Additionally, adjusting for BMI, smoking status, alcohol misuse and substance misuse did not alter the findings substantially (online supplemental table 7).

## Discussion

In this large, representative, longitudinal study, we found that multiple neurological conditions were more common in individuals with SMI than in a matched comparison group. However, there were important differences between conditions in both the strength and temporality of the relationship, facilitating a clearer understanding of the relationship between SMI and neurological disease. Focussing on the more prevalent neurological conditions, cerebrovascular disease and multiple sclerosis have a modest association with SMI, while the relationship with epilepsy, cerebral palsy and Parkinson’s disease is much stronger. The relationship between many neurological conditions and SMI is largely constant in the years prior to, during and after SMI diagnosis. However, for neurodegenerative conditions, such as dementia and Parkinson’s disease, the prevalence rises sharply in the years after SMI diagnosis. While compared with people without SMI, the prevalence of most neurological diseases was elevated in people with bipolar disorder and other psychoses; it was similar in people with schizophrenia.

Our approach had a number of strengths. The data set is large and has representative coverage from across the UK, which allows comparisons among rarer neurological conditions. Our follow-up period, which ended in 2018, avoids any disruption to diagnostic pathways caused by the COVID-19 pandemic. The precise diagnostic codes in CPRD allowed us to create specific categories that excluded conditions that might have different causal relationships (for instance, excluding other parkinsonism from the Parkinson’s disease category). Finally, our analysis strategy, adjusting for patient characteristics and then additionally health behaviours, allows comparison of different assumptions about the causal relationship between neurological conditions and SMI.

However, there are important limitations to this work. First, using electronic healthcare records relies on the accuracy of the diagnoses made in routine clinical care, which may be unstable. In our study, this may manifest in biases in either direction. On the one hand, patients with SMI have more contact with healthcare professionals, which may lead to more investigations and referrals for neurological conditions, resulting in surveillance bias. Conversely, diagnostic overshadowing—the tendency to attribute symptoms to a pre-existing condition—can result in patients with SMI having physical symptoms attributed to their mental health. Those with schizophrenia may be at particular risk of this, as for many neurological conditions, we found no evidence that prevalence rates in this group differed from the comparator group. The broad diagnostic codes used in primary care records may also have obscured some more subtle relationships, such as those described in the literature on ictal, post-ictal and interictal psychoses in epilepsy.

While we adjusted for several relevant covariates, there is likely to be some unmeasured confounding. Specifically, complications during gestation and birth have been associated with subsequent risk of both SMI and various neurological conditions, such as cerebral palsy, epilepsy and dementia. Conditioning on socioeconomic status is challenging in studies using healthcare records, which often lack a direct measure of this variable. Our analysis strategy aimed to handle this by matching based on primary care practice, but this is unlikely to fully capture socio-economic status in SMI, which seems to gradually decline over the life course and may not therefore be representative of living in a particular locality.

Despite the broad study population, there are challenges to its external validity. The age of the SMI cohort is higher than meta-analytic estimates of age of onset for schizophrenia (25), bipolar disorder (33) and other psychotic disorders (35).^25^ Given that CPRD is an accurate summary of primary care records, it is more likely that this result reflects delayed diagnosis or delayed recording of diagnosis.^9^ It is also possible that there is a delayed diagnosis of neurological conditions, so small changes in ORs over time should be interpreted with caution. The second challenge to external validity concerns the comparator cohort, which in matching based on variables to be more similar to the SMI cohort, is likely to be less healthy than the general population, as illustrated by the high morbidity and mortality of this group. Caution should be exercised when applying these results to populations outside the UK.

In aggregate, it seems reasonable to assume that—despite these limitations—various neurological conditions are overrepresented in individuals with SMI. Given our representative sample from primary care with very limited exclusions, it seems unlikely that collider stratification bias is responsible for this association. There are therefore four possible causal relationships between SMI and neurological conditions, although it is credible that different relationships exist for different conditions.

First, there is the possibility that SMI and some neurological conditions share a common cause. We have already mentioned the possibility of confounding by gestational factors or birth complications. Another plausible explanation is genetic factors. Genome-wide association studies have found a substantial overlap in genetic variants predisposing to psychiatric, neurodevelopmental, cognitive and other neurological conditions.^26 27^ It is plausible that such factors could result in the comorbidity of SMI and neurological conditions and that the age of onset might vary, accounting for some of the variability in relationships over time.

Second, it is possible that there is a psychiatric prodrome for some neurological conditions. In neurological conditions where the elevated odds in the SMI group are relatively constant over time, this seems unlikely, as a prodromal psychotic illness must precede frank clinical manifestations of the neurological disorder. However, dementia diagnosis rates only start to diverge substantially from the comparator group at the time of SMI diagnosis. When one considers that there may be a delay between the onset of symptoms of SMI and actual diagnosis, this seems to be a reasonable explanation. This is consistent with the existing literature suggesting that psychosis may be a common feature of prodromal dementia.^28^ The same relationship is possibly true to a lesser extent for Parkinson’s disease.

Third, it is possible that SMI causes neurological disorder. This seems unlikely for most disorders, where diagnosis rates are elevated consistently across the 10-year study period. If SMI causes neurological disorder, we would expect the association between them to increase after SMI diagnosis. In our data, one could support this hypothesis for dementia and Parkinson’s disease. However, this seems implausible based on our understanding of the pathophysiology of these conditions. In Parkinson’s disease, there is evidence that at the point of motor symptom onset, 68%–82% of striatal dopamine has already been lost^29^ and median time from motor onset to diagnosis is approximately 1 year.^30^ Similarly, in Alzheimer’s disease, there is evidence that markers of neurodegeneration are raised several years before symptomatic onset. It therefore seems unlikely that having an SMI diagnosis is actually causative of a neurodegenerative disease a few years later. An exception to this argument would be the other parkinsonism category, where there is a strong association with SMI, which becomes still stronger further after SMI diagnosis, a relationship that is potentially mediated by the effect of antipsychotic drugs. Psychotropic medications with an anti-cholinergic effect may contribute to diagnoses of dementia.

Fourth, it is possible that having a neurological condition causes SMI. There is evidence for a neurodevelopmental basis for psychotic disorders. Neurodevelopmental factors are risk factors that act in consort with other factors in later life, particularly in adolescence, in the genesis of SMI. Within this model, it is possible that the persistently elevated temporal relationship with neurological conditions starting in early life, such as cerebral palsy and structural developmental anomalies, is due to long-term effects of neurological conditions causing SMI.

In sum, genetic and gestational factors are a likely common cause of some neurological conditions and SMI; in neurodegenerative conditions, it is likely that there is an SMI prodrome; it is possible that SMI causes some neurological conditions (and antipsychotic medications may contribute to this association); and it is possible that neurological disorders with an early onset cause SMI.

### Conclusion

Regardless of causation, clinicians must be aware that neurological conditions and SMI are highly comorbid. Neurologists will encounter people with SMI, regardless of their neurological subspecialty. Psychiatrists should be aware that their patients are at risk of a wide range of neurological conditions, some of which may be undiagnosed at the point at which SMI is recognised. They should be particularly aware of the risk of incident dementia or Parkinson’s disease.

## Supplementary material

10.1136/bmjment-2025-301923 Supplementary file 1

## Data Availability

Data may be obtained from a third party and are not publicly available.
