# Supplementary material for: Temporal relationship between severe mental illness and neurological conditions in a UK primary care cohort
Source: BMJ Ment Health. 2025 Nov 4;28(1):e301923. doi: 10.1136/bmjment-2025-301923 (PMC12587905; doi:10.1136/bmjment-2025-301923)

### Supplementary Table 1: STROBE checklist

|  | Item No | Recommendation | Page number |
| --- | --- | --- | --- |
| **Title and abstract** | 1 | (*a*) Indicate the study’s design with a commonly used term in the title or the abstract | 1, 2 |
|  |  | (*b*) Provide in the abstract an informative and balanced summary of what was done and what was found | 2 |
| Introduction | | | |
| Background/rationale | 2 | Explain the scientific background and rationale for the investigation being reported | 4 |
| Objectives | 3 | State specific objectives, including any prespecified hypotheses | 4, 5 |
| Methods | | | |
| Study design | 4 | Present key elements of study design early in the paper | 5, 6 |
| Setting | 5 | Describe the setting, locations, and relevant dates, including periods of recruitment, exposure, follow-up, and data collection | 5 |
| Participants | 6 | (*a*) Give the eligibility criteria, and the sources and methods of selection of participants | 5 |
| Variables | 7 | Clearly define all outcomes, exposures, predictors, potential confounders, and effect modifiers. Give diagnostic criteria, if applicable | 5, 6 |
| Data sources/ measurement | 8* | For each variable of interest, give sources of data and details of methods of assessment (measurement). Describe comparability of assessment methods if there is more than one group | 5, 6 |
| Bias | 9 | Describe any efforts to address potential sources of bias | 5, 6 |
| Study size | 10 | Explain how the study size was arrived at | 5 |
| Quantitative variables | 11 | Explain how quantitative variables were handled in the analyses. If applicable, describe which groupings were chosen and why | 5, 6 |
| Statistical methods | 12 | (*a*) Describe all statistical methods, including those used to control for confounding | 6 |
|  |  | (*b*) Describe any methods used to examine subgroups and interactions | 6 |
|  |  | (*c*) Explain how missing data were addressed | 6 |
|  |  | (*d*) If applicable, describe analytical methods taking account of sampling strategy | N/A |
|  |  | (*e*) Describe any sensitivity analyses | 6 |
| Results | | | |
| Participants | 13* | (a) Report numbers of individuals at each stage of study—eg numbers potentially eligible, examined for eligibility, confirmed eligible, included in the study, completing follow-up, and analysed | 7, Supplementary Table 2 |
|  |  | (b) Give reasons for non-participation at each stage | Supplementary Table 2 |
|  |  | (c) Consider use of a flow diagram | N/A |
| Descriptive data | 14* | (a) Give characteristics of study participants (eg demographic, clinical, social) and information on exposures and potential confounders | 15 |
|  |  | (b) Indicate number of participants with missing data for each variable of interest | 15 |
| Outcome data | 15* | Report numbers of outcome events or summary measures | 16, 17 |
| Main results | 16 | (*a*) Give unadjusted estimates and, if applicable, confounder-adjusted estimates and their precision (eg, 95% confidence interval). Make clear which confounders were adjusted for and why they were included | 18 – 20, Supplementary Table 4 |
|  |  | (*b*) Report category boundaries when continuous variables were categorized | 6 |
|  |  | (*c*) If relevant, consider translating estimates of relative risk into absolute risk for a meaningful time period | N/A |
| Other analyses | 17 | Report other analyses done—eg analyses of subgroups and interactions, and sensitivity analyses | 21, 22, Supplementary Table 5 |
| Discussion | | | |
| Key results | 18 | Summarise key results with reference to study objectives | 7 |
| Limitations | 19 | Discuss limitations of the study, taking into account sources of potential bias or imprecision. Discuss both direction and magnitude of any potential bias | 8 |
| Interpretation | 20 | Give a cautious overall interpretation of results considering objectives, limitations, multiplicity of analyses, results from similar studies, and other relevant evidence | 9 |
| Generalisability | 21 | Discuss the generalisability (external validity) of the study results | 8 |
| Other information | | | |
| Funding | 22 | Give the source of funding and the role of the funders for the present study and, if applicable, for the original study on which the present article is based | 10 |

### Supplementary Table 2: Reasons for exclusions and corresponding numbers

|  | Number excluded | | Number remaining | |
| --- | --- | --- | --- | --- |
| Exclusion criteria | SMI | No SMI | SMI | No SMI |
| Original | NA | NA | 70855 | 283398 |
| Exited cohort before the start of follow up | 205 | 1208 | 70650 | 283295 |
| Entered cohort after the end of follow up | 71 | 266 | 70579 | 282572 |
| Diagnosed outside of 2000-2018 | 172 | NA | 70407 | 281924 |
| Diagnosed before 18 or over 100 | 1586 | NA | 68821 | 281924 |
| Starts over age 100 years or ends under 18 years | 0 | 7 | 68821 | 281917 |
| No region | 29 | 116 | 68792 | 281801 |
| Not active at GP surgery on case diagnosis date | 2 | 6194 | 68790 | 275607 |
| Unmatched | 1 | 780 | 68789 | 274827 |

SMI – severe mental illness

GP – general practitioner

### Supplementary Table 3: Neurological disease cumulative prevalence in people with severe mental illness and matched cohort

|  | **Years before SMI diagnosis** | | | | | |  | | **Years after SMI diagnosis** | | | | | |
| --- | --- | --- | --- | --- | --- | --- | --- | --- | --- | --- | --- | --- | --- | --- |
|  | **-5** | | **-3** | | **-1** | | **Index date** | | **1** | | **3** | | **5** | |
|  | SMI cohort (n= 68789) | Matched cohort  (n= 274827) | SMI cohort (n= 68789) | Matched cohort  (n= 274827) | SMI cohort (n= 68789) | Matched cohort  (n= 274827) | SMI cohort (n= 68789) | Matched cohort  (n= 274827) | SMI cohort (n= 68788) | Matched cohort  (n= 274731) | SMI cohort (n= 55647) | Matched cohort  (n= 209541) | SMI cohort (n= 38285) | Matched cohort  (n= 150733) |
| **Multiple Sclerosis (n, %)** | 149 (0.22) | 439 (0.16) | 174 (0.25) | 512 (0.19) | 204 (0.30) | 576 (0.21) | 219 (0.32) | 609 (0.22) | 251 (0.36) | 638 (0.23) | 222 (0.40) | 555 (0.26) | 156 (0.41) | 437 (0.29) |
| **Cerebrovascular disease (n, %)** | 983 (1.43) | 3454 (1.26) | 1278 (1.86) | 4421 (1.61) | 1698 (2.47) | 5604 (2.04) | 2079 (3.02) | 6302 (2.29) | 2472 (3.59) | 7037 (2.56) | 2237 (4.02) | 5977 (2.85) | 1620 (4.23) | 4681 (3.11) |
| **Dementia (n, %)** | 114 (0.17) | 405 (0.15) | 260 (0.38) | 817 (0.30) | 613 (0.89) | 1449 (0.53) | 1132 (1.65) | 1876 (0.68) | 2140 (3.11) | 2364 (0.86) | 1919 (3.45) | 1898 (0.91) | 1116 (2.91) | 1397 (0.93) |
| **Ataxia (n, %)** | 69 (0.10) | 130 (0.05) | 80 (0.12) | 164 (0.06) | 91 (0.13) | 200 (0.07) | 100 (0.15) | 209 (0.08) | 127 (0.18) | 224 (0.08) | 126 (0.23) | 182 (0.09) | 92 (0.24) | 143 (0.09) |
| **Epilepsy (n, %)** | 1788 (2.60) | 3368 (1.23) | 2013 (2.93) | 3696 (1.34) | 2289 (3.33) | 4102 (1.49) | 2553 (3.71) | 4352 (1.58) | 2980 (4.33) | 4541 (1.65) | 2692 (4.84) | 3669 (1.75) | 1990 (5.20) | 2717 (1.80) |
| **Parkinson's disease (n, %)** | 244 (0.35) | 319 (0.12) | 314 (0.46) | 409 (0.15) | 393 (0.57) | 393 (0.57) | 468 (0.68) | 645 (0.23) | 581 (0.84) | 733 (0.27) | 530 (0.95) | 594 (0.28) | 352 (0.92) | 418 (0.28) |
| **Paralysis (n, %)** | 174 (0.25) | 390 (0.14) | 191 (0.28) | 430 (0.16) | 204 (0.30) | 465 (0.17) | 218 (0.32) | 487 (0.18) | 239 (0.35) | 509 (0.19) | 220 (0.40) | 428 (0.20) | 144 (0.38) | 335 (0.22) |
| **Cerebral palsy (n, %)** | 157 (0.23) | 246 (0.09) | 159 (0.23) | 255 (0.09) | 162 (0.24) | 262 (0.10) | 168 (0.24) | 267 (0.10) | 176 (0.26) | 268 (0.10) | 146 (0.26) | 223 (0.11) | 100 (0.26) | 163 (0.11) |
| **CSF disorders (n, %)** | 60  (0.09) | 131 (0.05) | 63  (0.09) | 141 (0.05) | 70  (0.10) | 150 (0.05) | 77  (0.11) | 153 (0.06) | 95  (0.14) | 168 (0.06) | 86  (0.15) | 142 (0.07) | 62  (0.16) | 102 (0.07) |
| **Spinal cord disorders (n, %)** | 25  (0.04) | 77  (0.03) | 31  (0.05) | 91  (0.03) | 40  (0.06) | 111 (0.04) | 48  (0.07) | 121 (0.04) | 60  (0.09) | 142 (0.05) | 55  (0.10) | 125 (0.06) | 36  (0.09) | 100 (0.07) |
| **Parkinsonism (other) (n, %)** | 21  (0.03) | 11  (0.00) | 36  (0.05) | 15  (0.01) | 50  (0.07) | 32  (0.01) | 80  (0.12) | 38  (0.01) | 136 (0.20) | 51  (0.02) | 152 (0.27) | 42  (0.02) | 119 (0.31) | 28  (0.02) |
| **Nerve root, plexus & peripheral nervous system (n, %)** | 41  (0.06) | 118 (0.04) | 43  (0.06) | 135 (0.05) | 51  (0.07) | 146 (0.05) | 59  (0.09) | 155 (0.06) | 71  (0.10) | 166 (0.06) | 69  (0.12) | 136 (0.06) | 51  (0.13) | 105 (0.07) |
|  |  | | | | | |  | |  | | | | | |
|  | **Years before SMI diagnosis** | | | | | |  | | **Years after SMI diagnosis** | | | | | |
|  | **-5** | | **-3** | | **-1** | | **Index date** | | **1** | | **3** | | **5** | |
| **Movement disorders (other) (n, %)** | 26  (0.04) | 54  (0.02) | 34  (0.05) | 58  (0.02) | 43  (0.06) | 69  (0.03) | 53  (0.08) | 70  (0.03) | 72  (0.10) | 78  (0.03) | 66  (0.12) | 63  (0.03) | 50  (0.13) | 47  (0.03) |
| **Motor Neuron Disease (n, %)** | <5  (0.00) | 17  (0.01) | <5  (0.00) | 23  (0.01) | 5  (0.01) | 28  (0.01) | 5  (0.01) | 28  (0.01) | 12  (0.02) | 31  (0.01) | 10  (0.02) | 27  (0.01) | 6  (0.02) | 14  (0.01) |
| **Autonomic nervous system disorders (n, %)** | 8  (0.01) | 20  (0.01) | 10  (0.01) | 21  (0.01) | 10  (0.01) | 22  (0.01) | 11  (0.02) | 22  (0.01) | 11  (0.02) | 22  (0.01) | 11  (0.02) | 12  (0.01) | 8  (0.02) | 9  (0.01) |

### Supplementary Table 4: Medical codes

#### Severe Mental Illness codes

<https://phenotypes.healthdatagateway.org/phenotypes/PH1649/version/3407/detail/>

#### Neurological conditions codes

| **Term** | **Group** | **Umbrella** | **Original Read code** | **Cleansed Read code** |
| --- | --- | --- | --- | --- |
| [d]ataxia nos | Ataxic disorders | Ataxic disorders | R0130 | R013000 |
| [x]other hereditary ataxias | Ataxic disorders | Ataxic disorders | Fyu10 | Fyu1000 |
| ataxia-telangiectasia | Ataxic disorders | Ataxic disorders | F14y0 | F14y000 |
| cerebellar (nontraum) and post fossa haemorhage fet newborn | Ataxic disorders | Ataxic disorders | Q4171 | Q417100 |
| cerebellar abscess | Ataxic disorders | Ataxic disorders | F0401-1 | F040111 |
| cerebellar ataxia due to alcoholism | Ataxic disorders | Ataxic disorders | F1440 | F144000 |
| cerebellar ataxia in disease nos | Ataxic disorders | Ataxic disorders | F144z | F144z00 |
| cerebellar ataxia in diseases ec | Ataxic disorders | Ataxic disorders | F144 | F144.00 |
| cerebellar ataxia nos | Ataxic disorders | Ataxic disorders | F143 | F143.00 |
| cerebellar disease | Ataxic disorders | Ataxic disorders | F14-1 | F14..11 |
| cerebellar hypoplasia | Ataxic disorders | Ataxic disorders | P22z-1 | P22z.11 |
| cerebral ataxia | Ataxic disorders | Ataxic disorders | F11y1 | F11y100 |
| childhood cerebral degenerations nos | Ataxic disorders | Ataxic disorders | F10z | F10z.00 |
| fragile x associated tremor ataxia syndrome | Ataxic disorders | Ataxic disorders | EMISNQFR20 |  |
| fragile x associated tremor ataxia syndrome | Ataxic disorders | Ataxic disorders | F1y0 | F1y0.00 |
| friedreichs ataxia | Ataxic disorders | Ataxic disorders | F140 | F140.00 |
| locomotor ataxia | Ataxic disorders | Ataxic disorders | A940-1 | A940.11 |
| maries cerebellar ataxia | Ataxic disorders | Ataxic disorders | F1420 | F142000 |
| neuropathy in association with hereditary ataxia | Ataxic disorders | Ataxic disorders | F365 | F365.00 |
| other spinocerebellar disease nos | Ataxic disorders | Ataxic disorders | F14yz | F14yz00 |
| other spinocerebellar diseases | Ataxic disorders | Ataxic disorders | F14y | F14y.00 |
| primary cerebellar degeneration | Ataxic disorders | Ataxic disorders | F142 | F142.00 |
| primary cerebellar degeneration nos | Ataxic disorders | Ataxic disorders | F142z | F142z00 |
| spinocerebellar disease | Ataxic disorders | Ataxic disorders | F14 | F14..00 |
| spinocerebellar disease nos | Ataxic disorders | Ataxic disorders | F14z | F14z.00 |
| ataxia | Ataxic disorders | Ataxic disorders | NA | Z7E4.00 |
| cerebellar ataxia | Ataxic disorders | Ataxic disorders | NA | Z7E4400 |
| truncal ataxia | Ataxic disorders | Ataxic disorders | NA | Z7E4300 |
| [rfc] cerebral palsy | Cerebral palsy | Cerebral palsy | HNG0180 |  |
| [rfc] cerebral palsy | Cerebral palsy | Cerebral palsy | HNG0197 |  |
| [x]cerebral palsy and other paralytic syndromes | Cerebral palsy | Cerebral palsy | Fyu9 | Fyu9.00 |
| ataxic diplegic cerebral palsy | Cerebral palsy | Cerebral palsy | F23y4 | F23y400 |
| ataxic infantile cerebral palsy | Cerebral palsy | Cerebral palsy | F23y0 | F23y000 |
| athetoid cerebral palsy | Cerebral palsy | Cerebral palsy | F137-1 | F137.11 |
| athetoid cerebral palsy | Cerebral palsy | Cerebral palsy | F1370 | F137000 |
| cerebral palsy | Cerebral palsy | Cerebral palsy | F2B | F2B..00 |
| cerebral palsy nos | Cerebral palsy | Cerebral palsy | F2Bz | F2Bz.00 |
| cerebral palsy with spastic diplegia | Cerebral palsy | Cerebral palsy | F2301 | F230100 |
| cerebral palsy, not congenital or infantile, acute | Cerebral palsy | Cerebral palsy | G669 | G669.00 |
| cerebral palsy, not congenital or infantile, acute | Cerebral palsy | Cerebral palsy | G669 | G669.00 |
| choreoathetoid cerebral palsy | Cerebral palsy | Cerebral palsy | F23y6 | F23y600 |
| congenital cerebral palsy | Cerebral palsy | Cerebral palsy | F23 | F23..00 |
| congenital cerebral palsy nos | Cerebral palsy | Cerebral palsy | F23z | F23z.00 |
| congenital diplegia | Cerebral palsy | Cerebral palsy | F230 | F230.00 |
| congenital diplegia nos | Cerebral palsy | Cerebral palsy | F230z | F230z00 |
| congenital hemiplegia | Cerebral palsy | Cerebral palsy | F231 | F231.00 |
| congenital monoplegia | Cerebral palsy | Cerebral palsy | F233 | F233.00 |
| congenital paraplegia | Cerebral palsy | Cerebral palsy | F2300 | F230000 |
| congenital quadriplegia | Cerebral palsy | Cerebral palsy | F232 | F232.00 |
| congenital spastic cerebral palsy | Cerebral palsy | Cerebral palsy | F23-1 | F23..11 |
| dyskinetic cerebral palsy | Cerebral palsy | Cerebral palsy | F23y3 | F23y300 |
| flaccid infantile cerebral palsy | Cerebral palsy | Cerebral palsy | F23y1 | F23y100 |
| gross motor function classification system cerebral palsy | Cerebral palsy | Cerebral palsy | 38Gw | 38Gw.00 |
| infantile cerebral palsy | Cerebral palsy | Cerebral palsy | F23-2 | F23..12 |
| other cerebral palsy | Cerebral palsy | Cerebral palsy | F2By | F2By.00 |
| other infantile cerebral palsy nos | Cerebral palsy | Cerebral palsy | F23yz | F23yz00 |
| reason for referral: cerebral palsy | Cerebral palsy | Cerebral palsy | EMISNQRE209 |  |
| spastic cerebral palsy | Cerebral palsy | Cerebral palsy | F23y2 | F23y200 |
| spastic diplegic cerebral palsy | Cerebral palsy | Cerebral palsy | F2301-1 | F230111 |
| spastic hemiplegic cerebral palsy | Cerebral palsy | Cerebral palsy | F2B1 | F2B1.00 |
| spastic quadriplegic cerebral palsy | Cerebral palsy | Cerebral palsy | F2B0 | F2B0.00 |
| [rfc] cva | Cerebral ischaemia | Cerebrovascular disease | HNGP003 |  |
| [rfc] ischaemic attack | Cerebral ischaemia | Cerebrovascular disease | HNG0237 |  |
| [rfc] stroke | Cerebral ischaemia | Cerebrovascular disease | HNG0235 |  |
| [rfc] stroke | Cerebral ischaemia | Cerebrovascular disease | HNG0602 |  |
| [rfc] stroke/cva | Cerebral ischaemia | Cerebrovascular disease | HNG0234 |  |
| [v]personal history of cerebrovascular accident (cva) | Cerebral ischaemia | Cerebrovascular disease | ZV125-2 | ZV12512 |
| [v]personal history of stroke | Cerebral ischaemia | Cerebrovascular disease | ZV125-1 | ZV12511 |
| [x]cereb infarct due unsp occlus/stenos precerebr arteries | Cerebral ischaemia | Cerebrovascular disease | Gyu6G | Gyu6G00 |
| [x]cerebrl infarctn due/unspcf occlusn or sten/cerebrl artrs | Cerebral ischaemia | Cerebrovascular disease | Gyu63 | Gyu6300 |
| [x]cerebrovascular diseases | Cerebrovascular disease | Cerebrovascular disease | Gyu6 | Gyu6.00 |
| [x]intracerebral haemorrhage in hemisphere, unspecified | Intracranial haemorrhage | Cerebrovascular disease | Gyu6F | Gyu6F00 |
| [x]other cerebral infarction | Cerebral ischaemia | Cerebrovascular disease | Gyu64 | Gyu6400 |
| [x]other intracerebral haemorrhage | Intracranial haemorrhage | Cerebrovascular disease | Gyu62 | Gyu6200 |
| [x]other subarachnoid haemorrhage | Intracranial haemorrhage | Cerebrovascular disease | Gyu61 | Gyu6100 |
| [x]other vascular syndroms/brain in cerebrovasculr diseases | Cerebral ischaemia | Cerebrovascular disease | Fyu57 | Fyu5700 |
| [x]subarachnoid haemorrh from intracranial artery, unspecif | Intracranial haemorrhage | Cerebrovascular disease | Gyu6E | Gyu6E00 |
| [x]subarachnoid haemorrhage from other intracranial arteries | Intracranial haemorrhage | Cerebrovascular disease | Gyu60 | Gyu6000 |
| acute cerebrovascular insufficiency nos | Cerebral ischaemia | Cerebrovascular disease | G6710 | G671000 |
| acute confusional state, of cerebrovascular origin | Cerebrovascular disease | Cerebrovascular disease | E0304 | E030400 |
| acute confusional state, of cerebrovascular origin | Cerebral ischaemia | Cerebrovascular disease | E0304 | E030400 |
| admission to stroke unit | Cerebral ischaemia | Cerebrovascular disease | 8Hd6 | 8Hd6.00 |
| anoxic - ischaemic encephalopathy | Cerebrovascular disease | Cerebrovascular disease | F281-1 | F281.11 |
| basal nucleus haemorrhage | Intracranial haemorrhage | Cerebrovascular disease | G612 | G612.00 |
| basilar artery occlusion | Cerebral ischaemia | Cerebrovascular disease | G630 | G630.00 |
| basilar artery syndrome | TIA | Cerebrovascular disease | G650 | G650.00 |
| brain stem stroke syndrome | Vascular syndromes of brain in cerebrovascular diseases | Cerebrovascular disease | G663 | G663.00 |
| brainstem infarction | Cerebral ischaemia | Cerebrovascular disease | G64z0 | G64z000 |
| brainstem infarction nos | Cerebral ischaemia | Cerebrovascular disease | G64z-1 | G64z.11 |
| bulbar haemorrhage | Intracranial haemorrhage | Cerebrovascular disease | G615 | G615.00 |
| carotid artery disease | Cerebral ischaemia | Cerebrovascular disease | G70y0-1 | G70y011 |
| carotid artery doppler abnormal | Cerebral ischaemia | Cerebrovascular disease | 5C10 | 5C10.00 |
| carotid artery occlusion | Cerebral ischaemia | Cerebrovascular disease | G631 | G631.00 |
| carotid artery stenosis | Cerebral ischaemia | Cerebrovascular disease | G634 | G634.00 |
| carotid artery syndrome hemispheric | Cerebral ischaemia | Cerebrovascular disease | G653 | G653.00 |
| carotid endarterectomy and patch | Cerebral ischaemia | Cerebrovascular disease | 7A203-1 | 7A20311 |
| central post-stroke pain | Cerebral ischaemia | Cerebrovascular disease | 1M4 | 1M4..00 |
| central post-stroke pain | Cerebral ischaemia | Cerebrovascular disease | EMISNQCE10 |  |
| cereb infarct due cerebral venous thrombosis, nonpyogenic | Cerebral ischaemia | Cerebrovascular disease | G6760 | G676000 |
| cereb infarct due cerebral venous thrombosis, nonpyogenic | Cerebral ischaemia | Cerebrovascular disease | G6760 | G676000 |
| cereb infarct due unsp occlus/stenos precerebr arteries | Cerebral ischaemia | Cerebrovascular disease | G6W | G6W..00 |
| cerebellar haemorrhage | Intracranial haemorrhage | Cerebrovascular disease | G613 | G613.00 |
| cerebellar infarction | Cerebral ischaemia | Cerebrovascular disease | G64z-2 | G64z.12 |
| cerebellar stroke syndrome | Vascular syndromes of brain in cerebrovascular diseases | Cerebrovascular disease | G664 | G664.00 |
| cerebral arterial occlusion | Cerebral ischaemia | Cerebrovascular disease | G64 | G64..00 |
| cerebral degeneration due to cerebrovascular disease | Cerebral ischaemia | Cerebrovascular disease | F11x2 | F11x200 |
| cerebral degeneration due to multifocal leucoencephalopathy | Cerebral ischaemia | Cerebrovascular disease | F11x8 | F11x800 |
| cerebral degeneration in hunters disease | Cerebral ischaemia | Cerebrovascular disease | F1030 | F103000 |
| cerebral degeneration nos | Cerebral ischaemia | Cerebrovascular disease | F11z | F11z.00 |
| cerebral degenerations usually manifest in childhood | Cerebral ischaemia | Cerebrovascular disease | F10 | F10..00 |
| cerebral embolism | Cerebral ischaemia | Cerebrovascular disease | G641 | G641.00 |
| cerebral embolus | Cerebral ischaemia | Cerebrovascular disease | G641-1 | G641.11 |
| cerebral infarct due to thrombosis of precerebral arteries | Cerebral ischaemia | Cerebrovascular disease | G63y0 | G63y000 |
| cerebral infarction due to embolism of cerebral arteries | Cerebral ischaemia | Cerebrovascular disease | G6410 | G641000 |
| cerebral infarction due to embolism of precerebral arteries | Cerebral ischaemia | Cerebrovascular disease | G63y1 | G63y100 |
| cerebral infarction due to thrombosis of cerebral arteries | Cerebral ischaemia | Cerebrovascular disease | G6400 | G640000 |
| cerebral infarction nos | Cerebral ischaemia | Cerebrovascular disease | G64z | G64z.00 |
| cerebral thrombosis | Cerebral ischaemia | Cerebrovascular disease | G640 | G640.00 |
| cerebral venous thrombosis | Cerebral ischaemia | Cerebrovascular disease | EMISNQCE11 |  |
| cerebrl infarctn due/unspcf occlusn or sten/cerebrl artrs | Cerebral ischaemia | Cerebrovascular disease | G6X | G6X..00 |
| cerebrovascular accident care plan | Cerebral ischaemia | Cerebrovascular disease | EMISNQCE16 |  |
| cerebrovascular disease | Cerebrovascular disease | Cerebrovascular disease | G6 | G6...00 |
| cerebrovascular disease management | Cerebrovascular disease | Cerebrovascular disease | TESTPCE1 |  |
| cerebrovascular disease monitoring default | Cerebrovascular disease | Cerebrovascular disease | EMISNQCE29 |  |
| cerebrovascular disease nos | Cerebrovascular disease | Cerebrovascular disease | G6z | G6z..00 |
| cerebrovascular system anomalies | Cerebrovascular disease | Cerebrovascular disease | P7y0 | P7y0.00 |
| cortical haemorrhage | Intracranial haemorrhage | Cerebrovascular disease | G610 | G610.00 |
| cva - cerebral artery occlusion | Cerebral ischaemia | Cerebrovascular disease | G64-1 | G64..11 |
| cva - cerebrovascular accid due to intracerebral haemorrhage | Intracranial haemorrhage | Cerebrovascular disease | G61-1 | G61..11 |
| cva - cerebrovascular accident in the puerperium | Cerebral ischaemia | Cerebrovascular disease | L440-1 | L440.11 |
| cva - cerebrovascular accident unspecified | Cerebral ischaemia | Cerebrovascular disease | G66-3 | G66..13 |
| cva unspecified | Cerebral ischaemia | Cerebrovascular disease | G66-1 | G66..11 |
| delivery of rehabilitation for stroke | Cerebral ischaemia | Cerebrovascular disease | 7P242 | 7P24200 |
| discharge from community stroke service | Cerebral ischaemia | Cerebrovascular disease | EMISNQDI251 |  |
| endarterectomy and patch repair of carotid artery | Cerebral ischaemia | Cerebrovascular disease | 7A203 | 7A20300 |
| endarterectomy of carotid artery nec | Cerebral ischaemia | Cerebrovascular disease | 7A204 | 7A20400 |
| evacuation of intracerebral haematoma nec | Intracranial haemorrhage | Cerebrovascular disease | 70043 | 7004300 |
| external capsule haemorrhage | Intracranial haemorrhage | Cerebrovascular disease | G616 | G616.00 |
| generalised ischaemic cerebrovascular disease nos | Cerebral ischaemia | Cerebrovascular disease | G671 | G671.00 |
| generalised ischaemic cerebrovascular disease nos | Cerebral ischaemia | Cerebrovascular disease | G671z | G671z00 |
| h/o: cerebrovascular disease | Cerebrovascular disease | Cerebrovascular disease | 1477 | 1477 |
| h/o: cva/stroke | Cerebral ischaemia | Cerebrovascular disease | 14A7 | 14A7.00 |
| h/o: stroke | Cerebral ischaemia | Cerebrovascular disease | 14A7-2 | 14A7.12 |
| h/o: stroke in last year | Cerebral ischaemia | Cerebrovascular disease | 14AK | 14AK.00 |
| haemorrhagic stroke monitoring | Intracranial haemorrhage | Cerebrovascular disease | 662o | 662o.00 |
| infantile posthaemorrhagic hydrocephalus | Intracranial haemorrhage | Cerebrovascular disease | F117 | F117.00 |
| infarction - cerebral | Cerebral ischaemia | Cerebrovascular disease | G64-2 | G64..12 |
| infarction - precerebral | Cerebral ischaemia | Cerebrovascular disease | G63-1 | G63..11 |
| infarction of basal ganglia | Cerebral ischaemia | Cerebrovascular disease | G64z4 | G64z400 |
| insufficiency - basilar artery | Cerebral ischaemia | Cerebrovascular disease | G650-1 | G650.11 |
| intermittent cerebral ischaemia | Cerebral ischaemia | Cerebrovascular disease | G65z1 | G65z100 |
| internal capsule haemorrhage | Intracranial haemorrhage | Cerebrovascular disease | G611 | G611.00 |
| intracerebral (nontraumatic) haemorrhage of fet and newborn | Intracranial haemorrhage | Cerebrovascular disease | Q4170 | Q417000 |
| intracerebral haemorrhage | Intracranial haemorrhage | Cerebrovascular disease | G61 | G61..00 |
| intracerebral haemorrhage in fetus or newborn | Intracranial haemorrhage | Cerebrovascular disease | Q2000-1 | Q200011 |
| intracerebral haemorrhage in hemisphere, unspecified | Intracranial haemorrhage | Cerebrovascular disease | G61X | G61X.00 |
| intracerebral haemorrhage in hemisphere, unspecified | Intracranial haemorrhage | Cerebrovascular disease | G61X | G61X.00 |
| intracerebral haemorrhage nos | Intracranial haemorrhage | Cerebrovascular disease | G61z | G61z.00 |
| intracerebral haemorrhage, intraventricular | Intracranial haemorrhage | Cerebrovascular disease | G617 | G617.00 |
| intracerebral haemorrhage, intraventricular | Intracranial haemorrhage | Cerebrovascular disease | G617 | G617.00 |
| intracerebral haemorrhage, multiple localized | Intracranial haemorrhage | Cerebrovascular disease | G618 | G618.00 |
| intracranial haemorrhage nos | Intracranial haemorrhage | Cerebrovascular disease | G62z | G62z.00 |
| intraventric (nontraumatic) haemorrhage grade 4 fet newborn | Intracranial haemorrhage | Cerebrovascular disease | Q4114 |  |
| late effects of cerebrovascular disease | Cerebrovascular disease | Cerebrovascular disease | G68 | G68..00 |
| lateral medullary syndrome | Vascular syndromes of brain in cerebrovascular diseases | Cerebrovascular disease | G64z1-1 | G64z111 |
| left sided cerebral infarction | Cerebral ischaemia | Cerebrovascular disease | G64z2 | G64z200 |
| left sided cva | Cerebral ischaemia | Cerebrovascular disease | G667 | G667.00 |
| left sided intracerebral haemorrhage, unspecified | Intracranial haemorrhage | Cerebrovascular disease | G61X0 | G61X000 |
| left sided intracerebral haemorrhage, unspecified | Intracranial haemorrhage | Cerebrovascular disease | G61X0 | G61X000 |
| lobar cerebral haemorrhage | Intracranial haemorrhage | Cerebrovascular disease | G619 | G619.00 |
| middle cerebral artery syndrome | Vascular syndromes of brain in cerebrovascular diseases | Cerebrovascular disease | G660 | G660.00 |
| occlusion and stenosis of middle cerebral artery | Cerebral ischaemia | Cerebrovascular disease | G6770 | G677000 |
| occlusion/stenosis cerebral arts not result cerebral infarct | Cerebral ischaemia | Cerebrovascular disease | G677 | G677.00 |
| occlusive stroke | Cerebral ischaemia | Cerebrovascular disease | ESCTOC3 |  |
| other and unspecified intracranial haemorrhage | Intracranial haemorrhage | Cerebrovascular disease | G62 | G62..00 |
| other cerebral degeneration | Cerebral ischaemia | Cerebrovascular disease | F11y | F11y.00 |
| other cerebral degeneration nos | Cerebral ischaemia | Cerebrovascular disease | F11yz | F11yz00 |
| other cerebral degenerations | Cerebral ischaemia | Cerebrovascular disease | F11 | F11..00 |
| other cerebral haemorrhage following injury nos | Intracranial haemorrhage | Cerebrovascular disease | S63z | S63z.00 |
| other cerebrovascular disease | Cerebrovascular disease | Cerebrovascular disease | G67 | G67..00 |
| other cerebrovascular disease nos | Cerebrovascular disease | Cerebrovascular disease | G67z | G67z.00 |
| other cerebrovascular disease os | Cerebrovascular disease | Cerebrovascular disease | G67y | G67y.00 |
| other precerebral artery occlusion | Cerebral ischaemia | Cerebrovascular disease | G63y | G63y.00 |
| other specified cerebrovascular anomaly | Cerebrovascular disease | Cerebrovascular disease | P7y0y | P7y0y00 |
| other specified cerebrovascular disease | Cerebrovascular disease | Cerebrovascular disease | G6y | G6y..00 |
| other transient cerebral ischaemia | Cerebral ischaemia | Cerebrovascular disease | G65y | G65y.00 |
| pontine haemorrhage | Intracranial haemorrhage | Cerebrovascular disease | G614 | G614.00 |
| posterior cerebral artery syndrome | Vascular syndromes of brain in cerebrovascular diseases | Cerebrovascular disease | G662 | G662.00 |
| precerebral arterial occlusion | Cerebral ischaemia | Cerebrovascular disease | G63 | G63..00 |
| precerebral artery occlusion nos | Cerebral ischaemia | Cerebrovascular disease | G63z | G63z.00 |
| puerperal cerebrovascular disorder - delivered | Cerebrovascular disease | Cerebrovascular disease | L4401 | L440100 |
| puerperal cerebrovascular disorder unspecified | Cerebrovascular disease | Cerebrovascular disease | L4400 | L440000 |
| puerperal cerebrovascular disorder with antenatal comp | Cerebrovascular disease | Cerebrovascular disease | L4403 | L440300 |
| pure motor lacunar syndrome | Vascular syndromes of brain in cerebrovascular diseases | Cerebrovascular disease | G665 | G665.00 |
| pure sensory lacunar syndrome | Vascular syndromes of brain in cerebrovascular diseases | Cerebrovascular disease | G666 | G666.00 |
| ref multidisciplinary stroke function improvement declined | Cerebral ischaemia | Cerebrovascular disease | 8IEC | 8IEC.00 |
| ref to multidisciplinary stroke function improvement service | Cerebral ischaemia | Cerebrovascular disease | 8HHM | 8HHM.00 |
| referral by stroke nurse specialist | Cerebral ischaemia | Cerebrovascular disease | EMISNQRE381 |  |
| referral to community stroke service | Cerebral ischaemia | Cerebrovascular disease | EMISNQRE623 |  |
| referral to stroke rehabilitation service | Cerebral ischaemia | Cerebrovascular disease | EMISNQRE602 |  |
| right sided cerebral infarction | Cerebral ischaemia | Cerebrovascular disease | G64z3 | G64z300 |
| right sided cva | Cerebral ischaemia | Cerebrovascular disease | G668 | G668.00 |
| right sided intracerebral haemorrhage, unspecified | Intracranial haemorrhage | Cerebrovascular disease | G61X1 | G61X100 |
| right sided intracerebral haemorrhage, unspecified | Intracranial haemorrhage | Cerebrovascular disease | G61X1 | G61X100 |
| ruptured berry aneurysm | Cerebral ischaemia | Cerebrovascular disease | G600 | G600.00 |
| sequelae of cerebral infarction | Cerebral ischaemia | Cerebrovascular disease | G683 | G683.00 |
| sequelae of intracerebral haemorrhage | Intracranial haemorrhage | Cerebrovascular disease | G681 | G681.00 |
| sequelae of other nontraumatic intracranial haemorrhage | Intracranial haemorrhage | Cerebrovascular disease | G682 | G682.00 |
| sequelae of stroke,not specfd as hmorrhage or infarction | Cerebral ischaemia | Cerebrovascular disease | G68X | G68X.00 |
| sequelae of stroke,not specfd as hmorrhage or infarction | Cerebral ischaemia | Cerebrovascular disease | G68X | G68X.00 |
| sequelae of subarachnoid haemorrhage | Intracranial haemorrhage | Cerebrovascular disease | G680 | G680.00 |
| sequelae/other + unspecified cerebrovascular diseases | Cerebrovascular disease | Cerebrovascular disease | G68W | G68W.00 |
| small vessel cerebrovascular disease | Cerebral ischaemia | Cerebrovascular disease | G679 | G679.00 |
| stenosis of precerebral arteries | Cerebral ischaemia | Cerebrovascular disease | G63-2 | G63..12 |
| stenosis, carotid artery | Cerebral ischaemia | Cerebrovascular disease | G631-1 | G631.11 |
| stenosis, carotid artery | Cerebral ischaemia | Cerebrovascular disease | G631-1 | G631.11 |
| stroke / transient ischaemic attack referral | Cerebral ischaemia | Cerebrovascular disease | 8HBJ | 8HBJ.00 |
| stroke 6 month review | Cerebral ischaemia | Cerebrovascular disease | 662M1 | 662M100 |
| stroke and cerebrovascular accident unspecified | Cerebral ischaemia | Cerebrovascular disease | G66 | G66..00 |
| stroke annual review | Cerebral ischaemia | Cerebrovascular disease | 662e-1 | 662e.11 |
| stroke due to cerebral arterial occlusion | Cerebral ischaemia | Cerebrovascular disease | G64-3 | G64..13 |
| stroke due to intracerebral haemorrhage | Intracranial haemorrhage | Cerebrovascular disease | G61-2 | G61..12 |
| stroke group member | Cerebral ischaemia | Cerebrovascular disease | 13YA | 13YA.00 |
| stroke in the puerperium | Cerebral ischaemia | Cerebrovascular disease | L440-2 | L440.12 |
| stroke monitoring | Cerebral ischaemia | Cerebrovascular disease | 662M | 662M.00 |
| stroke monitoring invite 1 | Cerebral ischaemia | Cerebrovascular disease | EMISNQST2 |  |
| stroke monitoring invite 2 | Cerebral ischaemia | Cerebrovascular disease | EMISNQST3 |  |
| stroke monitoring invite 3 | Cerebral ischaemia | Cerebrovascular disease | EMISNQST4 |  |
| stroke self-management plan agreed | Cerebral ischaemia | Cerebrovascular disease | 661M7 | 661M700 |
| stroke unspecified | Cerebral ischaemia | Cerebrovascular disease | G66-2 | G66..12 |
| stroke/cva annual review | Cerebral ischaemia | Cerebrovascular disease | 662e | 662e.00 |
| stroke/transient ischaemic attack monitoring administration | Cerebral ischaemia | Cerebrovascular disease | 9Om | 9Om..00 |
| stroke/transient ischaemic attack monitoring first letter | Cerebral ischaemia | Cerebrovascular disease | 9Om0 | 9Om0.00 |
| stroke/transient ischaemic attack monitoring second letter | Cerebral ischaemia | Cerebrovascular disease | 9Om1 | 9Om1.00 |
| stroke/transient ischaemic attack monitoring third letter | Cerebral ischaemia | Cerebrovascular disease | 9Om2 | 9Om2.00 |
| stroke/transient ischaemic attack monitoring verbal invitati | Cerebral ischaemia | Cerebrovascular disease | 9Om3 | 9Om3.00 |
| subacute confusional state, of cerebrovascular origin | Cerebrovascular disease | Cerebrovascular disease | E0314 | E031400 |
| subacute confusional state, of cerebrovascular origin | Cerebrovascular disease | Cerebrovascular disease | E0314 | E031400 |
| subarachnoid haemorrh from intracranial artery, unspecif | Intracranial haemorrhage | Cerebrovascular disease | G60X | G60X.00 |
| subarachnoid haemorrh from intracranial artery, unspecif | Intracranial haemorrhage | Cerebrovascular disease | G60X | G60X.00 |
| subarachnoid haemorrhage | Intracranial haemorrhage | Cerebrovascular disease | G60 | G60..00 |
| subarachnoid haemorrhage from anterior communicating artery | Intracranial haemorrhage | Cerebrovascular disease | G603 | G603.00 |
| subarachnoid haemorrhage from basilar artery | Intracranial haemorrhage | Cerebrovascular disease | G605 | G605.00 |
| subarachnoid haemorrhage from carotid siphon and bifurcation | Intracranial haemorrhage | Cerebrovascular disease | G601 | G601.00 |
| subarachnoid haemorrhage from middle cerebral artery | Intracranial haemorrhage | Cerebrovascular disease | G602 | G602.00 |
| subarachnoid haemorrhage from posterior communicating artery | Intracranial haemorrhage | Cerebrovascular disease | G604 | G604.00 |
| subarachnoid haemorrhage from vertebral artery | Intracranial haemorrhage | Cerebrovascular disease | G606 | G606.00 |
| subarachnoid haemorrhage nos | Intracranial haemorrhage | Cerebrovascular disease | G60z | G60z.00 |
| subdural haemorrhage - nontraumatic | Intracranial haemorrhage | Cerebrovascular disease | G621 | G621.00 |
| subdural haemorrhage nos | Intracranial haemorrhage | Cerebrovascular disease | G623 | G623.00 |
| suspected cerebrovascular disease | Cerebrovascular disease | Cerebrovascular disease | 1JA1 | 1JA1.00 |
| suspected stroke | Cerebral ischaemia | Cerebrovascular disease | 1JA10-1 | 1JA1011 |
| suspected stroke | Cerebral ischaemia | Cerebrovascular disease | EMISNQSU28 |  |
| suspected transient ischaemic attack | Cerebral ischaemia | Cerebrovascular disease | 1JK | 1JK..00 |
| suspected transient ischaemic attack | Cerebral ischaemia | Cerebrovascular disease | EMISNQSU26 |  |
| tia clinical management plan no longer in place | Cerebral ischaemia | Cerebrovascular disease | EMISNQTI19 |  |
| transient cerebral ischaemia | Cerebral ischaemia | Cerebrovascular disease | G65 | G65..00 |
| transient cerebral ischaemia nos | Cerebral ischaemia | Cerebrovascular disease | G65z | G65z.00 |
| transient cerebral ischaemia nos | Cerebral ischaemia | Cerebrovascular disease | G65zz | G65zz00 |
| transient ischaemic attack | Cerebral ischaemia | Cerebrovascular disease | G65-2 | G65..12 |
| transient ischaemic attack clinical management plan | Cerebral ischaemia | Cerebrovascular disease | 8CRB | 8CRB.00 |
| transient ischaemic attack monitoring | Cerebral ischaemia | Cerebrovascular disease | EMISNQTR28 |  |
| traumatic subdural haemorrhage | Intracranial haemorrhage | Cerebrovascular disease | S628 | S628.00 |
| vertebral artery occlusion | Cerebral ischaemia | Cerebrovascular disease | G632 | G632.00 |
| vertebral artery syndrome | Cerebral ischaemia | Cerebrovascular disease | G651 | G651.00 |
| vertebro-basilar artery syndrome | Cerebral ischaemia | Cerebrovascular disease | G6510 | G651000 |
| vertebro-basilar insufficiency | Cerebral ischaemia | Cerebrovascular disease | G65-3 | G65..13 |
| vertebrobasilar insufficiency | Cerebral ischaemia | Cerebrovascular disease | G656 | G656.00 |
| wallenberg syndrome | Vascular syndromes of brain in cerebrovascular diseases | Cerebrovascular disease | G64z1 | G64z100 |
| [d] dementia | Dementia unknown/unspecified/other | Dementia | EMISNQDD2 |  |
| [d] dementia in alzheimers disease | Alzheimer's disease | Dementia | EMISNQDD3 |  |
| [d] dementia with lewy bodies | Lewy body dementia | Dementia | EMISNQDD1 |  |
| [d] vascular dementia | Vascular dementia | Dementia | EMISNQDV1 |  |
| [rfc] alzheimers disease | Alzheimer's disease | Dementia | HNG0062 |  |
| [rfc] dementia | Dementia unknown/unspecified/other | Dementia | HNGNQRF130 |  |
| [x] presenile dementia nos | Dementia unknown/unspecified/other | Dementia | Eu02z-1 | Eu02z11 |
| [x] primary degenerative dementia nos | Dementia unknown/unspecified/other | Dementia | Eu02z-3 | Eu02z13 |
| [x] senile dementia nos | Dementia unknown/unspecified/other | Dementia | Eu02z-4 | Eu02z14 |
| [x] senile dementia, depressed or paranoid type | Dementia unknown/unspecified/other | Dementia | Eu02z-6 | Eu02z16 |
| [x] senile dementia, depressed or paranoid type | Dementia unknown/unspecified/other | Dementia | Eu02z-6 | Eu02z16 |
| [x] unspecified dementia | Dementia unknown/unspecified/other | Dementia | Eu02z | Eu02z00 |
| [x]alzheimers dementia unspec | Alzheimer's disease | Dementia | Eu00z-1 | Eu00z11 |
| [x]alzheimers disease type 1 | Alzheimer's disease | Dementia | Eu001-1 | Eu00111 |
| [x]arteriosclerotic dementia | Cerebrovascular dementia | Dementia | Eu01-1 | Eu01.11 |
| [x]delirium superimposed on dementia | Dementia unknown/unspecified/other | Dementia | Eu041 | Eu04100 |
| [x]dementia in alzheimers dis, atypical or mixed type | Alzheimer's disease | Dementia | Eu002 | Eu00200 |
| [x]dementia in alzheimers dis, atypical or mixed type | Alzheimer's disease | Dementia | Eu002 | Eu00200 |
| [x]dementia in alzheimers disease | Alzheimer's disease | Dementia | Eu00 | Eu00.00 |
| [x]dementia in alzheimers disease with early onset | Alzheimer's disease | Dementia | Eu000 | Eu00000 |
| [x]dementia in alzheimers disease with late onset | Alzheimer's disease | Dementia | Eu001 | Eu00100 |
| [x]dementia in alzheimers disease, unspecified | Alzheimer's disease | Dementia | Eu00z | Eu00z00 |
| [x]dementia in alzheimers disease, unspecified | Alzheimer's disease | Dementia | Eu00z | Eu00z00 |
| [x]dementia in human immunodef virus [hiv] disease | Dementia unknown/unspecified/other | Dementia | Eu024 | Eu02400 |
| [x]dementia in huntingtons disease | Dementia unknown/unspecified/other | Dementia | Eu022 | Eu02200 |
| [x]dementia in other diseases classified elsewhere | Dementia unknown/unspecified | Dementia | Eu02 | Eu02.00 |
| [x]dementia in other specified diseases classif elsewhere | Dementia unknown/unspecified | Dementia | Eu02y | Eu02y00 |
| [x]dementia in picks disease | Dementia unknown/unspecified/other | Dementia | Eu020 | Eu02000 |
| [x]lewy body dementia | Lewy body dementia | Dementia | Eu025 | Eu02500 |
| [x]mixed cortical and subcortical vascular dementia | Vascular dementia | Dementia | Eu013 | Eu01300 |
| [x]multi-infarct dementia | Vascular dementia | Dementia | Eu011 | Eu01100 |
| [x]other alzheimers disease | Alzheimer's disease | Dementia | Fyu30 | Fyu3000 |
| [x]other vascular dementia | Vascular dementia | Dementia | Eu01y | Eu01y00 |
| [x]predominantly cortical dementia | Dementia unknown/unspecified/other | Dementia | Eu011-1 | Eu01111 |
| [x]presenile dementia,alzheimers type | Alzheimer's disease | Dementia | Eu000-1 | Eu00011 |
| [x]presenile dementia,alzheimers type | Alzheimer's disease | Dementia | Eu000-1 | Eu00011 |
| [x]primary degen dementia of alzheimers type, senile onset | Alzheimer's disease | Dementia | Eu001-3 | Eu00113 |
| [x]primary degen dementia of alzheimers type, senile onset | Alzheimer's disease | Dementia | Eu001-3 | Eu00113 |
| [x]primary degen dementia, alzheimers type, presenile onset | Alzheimer's disease | Dementia | Eu000-2 | Eu00012 |
| [x]senile dementia,alzheimers type | Alzheimer's disease | Dementia | Eu001-2 | Eu00112 |
| [x]senile dementia,alzheimers type | Alzheimer's disease | Dementia | Eu001-2 | Eu00112 |
| [x]subcortical vascular dementia | Vascular dementia | Dementia | Eu012 | Eu01200 |
| [x]vascular dementia | Vascular dementia | Dementia | Eu01 | Eu01.00 |
| [x]vascular dementia of acute onset | Vascular dementia | Dementia | Eu010 | Eu01000 |
| [x]vascular dementia, unspecified | Vascular dementia | Dementia | Eu01z | Eu01z00 |
| [x]vascular dementia, unspecified | Vascular dementia | Dementia | Eu01z | Eu01z00 |
| alzheimers disease | Alzheimer's disease | Dementia | F110 | F110.00 |
| alzheimers disease with early onset | Alzheimer's disease | Dementia | F1100 | F110000 |
| alzheimers disease with late onset | Alzheimer's disease | Dementia | F1101 | F110100 |
| antipsychotic drug therapy for dementia | Dementia unknown/unspecified/other | Dementia | 8BPa | 8BPa.00 |
| arteriosclerotic dementia | Vascular dementia | Dementia | E004 | E004.00 |
| arteriosclerotic dementia nos | Vascular dementia | Dementia | E004z | E004z00 |
| arteriosclerotic dementia with delirium | Vascular dementia | Dementia | E0041 | E004100 |
| arteriosclerotic dementia with paranoia | Vascular dementia | Dementia | E0042 | E004200 |
| dementia care plan | Dementia unknown/unspecified/other | Dementia | EMISNQDE37 |  |
| dementia care plan | Dementia unknown/unspecified/other | Dementia | 8CMZ | 8CMZ.00 |
| dementia confirmed | Dementia unknown/unspecified | Dementia | EMISNQDE35 |  |
| dementia in alzheimers dis, atypical or mixed type, other mixed symptoms | Alzheimer's disease | Dementia | EMISICD10\|F0024 | |
| dementia in conditions ec | Dementia unknown/unspecified/other | Dementia | E041 | E041.00 |
| dementia link worker - mental health cpn | Dementia unknown/unspecified/other | Dementia | EMISNQDE117 |  |
| dementia monitoring | Dementia unknown/unspecified/other | Dementia | 66h | 66h..00 |
| dementia monitoring administration | Dementia unknown/unspecified/other | Dementia | 9Ou | 9Ou..00 |
| dementia monitoring first letter | Dementia unknown/unspecified/other | Dementia | 9Ou1 | 9Ou1.00 |
| dementia monitoring in primary care | Dementia unknown/unspecified/other | Dementia | EMISNQDE41 |  |
| dementia monitoring in secondary care | Dementia unknown/unspecified/other | Dementia | EMISNQDE42 |  |
| dementia monitoring second letter | Dementia unknown/unspecified/other | Dementia | 9Ou2 | 9Ou2.00 |
| dementia monitoring third letter | Dementia unknown/unspecified/other | Dementia | 9Ou3 | 9Ou3.00 |
| dementia monitoring verbal invite | Dementia unknown/unspecified/other | Dementia | 9Ou4 | 9Ou4.00 |
| dementia review | Dementia unknown/unspecified/other | Dementia | EMISNQDE1 |  |
| dementia review with third party | Dementia unknown/unspecified/other | Dementia | EMISNQDE2 |  |
| dementia stage at diagnosis - early (mild) | Dementia unknown/unspecified/other | Dementia | EMISNQDE68 |  |
| dementia stage at diagnosis - late (severe) | Dementia unknown/unspecified/other | Dementia | EMISNQDE70 |  |
| dementia stage at diagnosis - mid (moderate) | Dementia unknown/unspecified/other | Dementia | EMISNQDE69 |  |
| did not attend dementia monitoring | Dementia unknown/unspecified/other | Dementia | EMISNQDI117 |  |
| drug-induced dementia | Dementia unknown/unspecified/other | Dementia | E02y1 | E02y100 |
| h/o: dementia | Dementia unknown/unspecified/other | Dementia | 1461 | 1461 |
| history of creutzfeldt-jakob disease | Human prion disorders | Dementia | JHCHI32 |  |
| history of suspected creutzfeldt-jakob disease | Human prion disorders | Dementia | JHCHI33 |  |
| jakob-creutzfeldt disease | Human prion disorders | Dementia | A411 | A411.00 |
| local alzheimers society involved | Alzheimer's disease | Dementia | EMISNQEX1 |  |
| mixed cortical and subcortical vascular dementia, other mixed symptoms | Vascular dementia | Dementia | EMISICD10\|F0134 | |
| mixed cortical and subcortical vascular dementia, other symptoms, predominantly delusional | Vascular dementia | Dementia | EMISICD10\|F0131 | |
| mixed cortical and subcortical vascular dementia, other symptoms, predominantly depressive | Vascular dementia | Dementia | EMISICD10\|F0133 | |
| mixed cortical and subcortical vascular dementia, other symptoms, predominantly hallucinatory | Vascular dementia | Dementia | EMISICD10\|F0132 | |
| mixed cortical and subcortical vascular dementia, without additional symptoms | Vascular dementia | Dementia | EMISICD10\|F0130 | |
| multi infarct dementia | Vascular dementia | Dementia | E004-1 | E004.11 |
| other alcoholic dementia | Dementia unknown/unspecified/other | Dementia | E012 | E012.00 |
| other vascular dementia, other mixed symptoms | Vascular dementia | Dementia | EMISICD10\|F0184 | |
| other vascular dementia, other symptoms, predominantly delusional | Vascular dementia | Dementia | EMISICD10\|F0181 | |
| other vascular dementia, other symptoms, predominantly depressive | Vascular dementia | Dementia | EMISICD10\|F0183 | |
| other vascular dementia, other symptoms, predominantly hallucinatory | Vascular dementia | Dementia | EMISICD10\|F0182 | |
| other vascular dementia, without additional symptoms | Vascular dementia | Dementia | EMISICD10\|F0180 | |
| person centred dementia support plan (scotland) | Dementia unknown/unspecified/other | Dementia | EMISNQPE63 |  |
| presenile dementia | Dementia unknown/unspecified/other | Dementia | E001 | E001.00 |
| presenile dementia nos | Dementia unknown/unspecified/other | Dementia | E001z | E001z00 |
| presenile dementia with delirium | Dementia unknown/unspecified/other | Dementia | E0011 | E001100 |
| presenile dementia with depression | Dementia unknown/unspecified/other | Dementia | E0013 | E001300 |
| presenile dementia with paranoia | Dementia unknown/unspecified/other | Dementia | E0012 | E001200 |
| refer to dementia care advisor | Dementia unknown/unspecified/other | Dementia | EMISNQRE154 |  |
| referral to dementia care advisor | Dementia unknown/unspecified/other | Dementia | 8Hla | 8Hla.00 |
| referral to dementia early intervention service | Dementia unknown/unspecified/other | Dementia | EMISNQRE491 |  |
| senile dementia | Dementia unknown/unspecified/other | Dementia | E00-1 | E00..11 |
| senile dementia with delirium | Dementia unknown/unspecified/other | Dementia | E003 | E003.00 |
| senile dementia with depression | Dementia unknown/unspecified/other | Dementia | E0021 | E002100 |
| senile dementia with depressive or paranoid features | Dementia unknown/unspecified/other | Dementia | E002 | E002.00 |
| senile dementia with depressive or paranoid features nos | Dementia unknown/unspecified/other | Dementia | E002z | E002z00 |
| senile dementia with paranoia | Dementia unknown/unspecified/other | Dementia | E0020 | E002000 |
| senile/presenile dementia | Dementia unknown/unspecified/other | Dementia | E00-2 | E00..12 |
| shared care prescribing of drugs for dementia | Dementia unknown/unspecified/other | Dementia | 8BM50 |  |
| shared care prescribing of drugs for dementia declined | Dementia unknown/unspecified/other | Dementia | 8BM60 |  |
| signposting to dementia support service | Dementia unknown/unspecified/other | Dementia | ESCTSI53 |  |
| subcortical vascular dementia, other mixed symptoms | Vascular dementia | Dementia | EMISICD10\|F0124 | |
| subcortical vascular dementia, other symptoms, predominantly delusional | Vascular dementia | Dementia | EMISICD10\|F0121 | |
| subcortical vascular dementia, other symptoms, predominantly depressive | Vascular dementia | Dementia | EMISICD10\|F0123 | |
| subcortical vascular dementia, other symptoms, predominantly hallucinatory | Vascular dementia | Dementia | EMISICD10\|F0122 | |
| subcortical vascular dementia, without additional symptoms | Vascular dementia | Dementia | EMISICD10\|F0120 | |
| uncomplicated arteriosclerotic dementia | Vascular dementia | Dementia | E0040 | E004000 |
| uncomplicated presenile dementia | Dementia unknown/unspecified/other | Dementia | E0010 | E001000 |
| uncomplicated senile dementia | Dementia unknown/unspecified/other | Dementia | E000 | E000.00 |
| unspecified dementia, other mixed symptoms | Dementia unknown/unspecified/other | Dementia | EMISICD10\|F03X4 | |
| unspecified dementia, other symptoms, predominantly delusional | Dementia unknown/unspecified/other | Dementia | EMISICD10\|F03X1 | |
| unspecified dementia, other symptoms, predominantly depressive | Dementia unknown/unspecified/other | Dementia | EMISICD10\|F03X3 | |
| unspecified dementia, other symptoms, predominantly hallucinatory | Dementia unknown/unspecified/other | Dementia | EMISICD10\|F03X2 | |
| unspecified dementia, without additional symptoms | Dementia unknown/unspecified/other | Dementia | EMISICD10\|F03X0 | |
| variant creutzfeldt-jakob disease | Human prion disorders | Dementia | EMISNQVA13 |  |
| vascular dementia of acute onset, other mixed symptoms | Vascular dementia | Dementia | EMISICD10\|F0104 | |
| vascular dementia of acute onset, other symptoms, predominantly delusional | Vascular dementia | Dementia | EMISICD10\|F0101 | |
| vascular dementia of acute onset, other symptoms, predominantly depressive | Vascular dementia | Dementia | EMISICD10\|F0103 | |
| vascular dementia of acute onset, other symptoms, predominantly hallucinatory | Vascular dementia | Dementia | EMISICD10\|F0102 | |
| vascular dementia of acute onset, without additional symptoms | Vascular dementia | Dementia | EMISICD10\|F0100 | |
| vascular dementia, unspecified, other mixed symptoms | Vascular dementia | Dementia | EMISICD10\|F0194 | |
| vascular dementia, unspecified, other symptoms, predominantly delusional | Vascular dementia | Dementia | EMISICD10\|F0191 | |
| vascular dementia, unspecified, other symptoms, predominantly depressive | Vascular dementia | Dementia | EMISICD10\|F0193 | |
| vascular dementia, unspecified, other symptoms, predominantly hallucinatory | Vascular dementia | Dementia | EMISICD10\|F0192 | |
| vascular dementia, unspecified, without additional symptoms | Vascular dementia | Dementia | EMISICD10\|F0190 | |
| [d]reflex anoxic seizure | Reflex syncope | Disorders of autonomic nervous system | R0033 | R003300 |
| multiple system atrophy, cerebellar variant | Disorders of autonomic nervous system | Disorders of autonomic nervous system | F1740 |  |
| multiple system atrophy, parkinson variant | Disorders of autonomic nervous system | Disorders of autonomic nervous system | F1741 |  |
| acute radial nerve palsy | Mononeuropathy | Disorders of nerve root, plexus or peripheral nerves | F3430 | F343000 |
| brachial plexus palsy due to birth trauma | Mononeuropathy | Disorders of nerve root, plexus or peripheral nerves | Q206 | Q206.00 |
| correction of erbs palsy | Mononeuropathy | Disorders of nerve root, plexus or peripheral nerves | 7L0D1-1 | 7L0D111 |
| erb-duchenne paralysis | Mononeuropathy | Disorders of nerve root, plexus or peripheral nerves | Q2061-1 | Q206111 |
| erbs palsy | Mononeuropathy | Disorders of nerve root, plexus or peripheral nerves | Q2061-2 | Q206112 |
| lepiscopo operation for obstetric palsy | Mononeuropathy | Disorders of nerve root, plexus or peripheral nerves | 7L0D1-4 | 7L0D114 |
| other specified brachial plexus palsy due to birth trauma | Mononeuropathy | Disorders of nerve root, plexus or peripheral nerves | Q206y | Q206y00 |
| peroneal muscular atrophy | Hereditary neuropathy | Disorders of nerve root, plexus or peripheral nerves | F361 | F361.00 |
| polyneuropathy in beriberi | Polyneuropathy | Disorders of nerve root, plexus or peripheral nerves | F3741 | F374100 |
| sever operation for erbs palsy | Mononeuropathy | Disorders of nerve root, plexus or peripheral nerves | 7L0D1-5 | 7L0D115 |
| specified palsy nec | Mononeuropathy | Disorders of nerve root, plexus or peripheral nerves | F24yz-1 | F24yz11 |
| tardy ulnar nerve palsy | Mononeuropathy | Disorders of nerve root, plexus or peripheral nerves | F3421 | F342100 |
| [d]nocturnal seizure | Epilepsy | Epilepsy or seizures | R0034 | R003400 |
| [d]seizure nos | Epilepsy | Epilepsy or seizures | R003z-1 | R003z11 |
| [rfc] epilepsy/convulsion | Epilepsy | Epilepsy or seizures | HNG0067 |  |
| [x]acquired aphasia with epilepsy [landau - kleffner] | Epilepsy | Epilepsy or seizures | Eu803 | Eu80300 |
| [x]epileptic psychosis nos | Epilepsy | Epilepsy or seizures | Eu05y-1 | Eu05y11 |
| [x]limbic epilepsy personality | Epilepsy | Epilepsy or seizures | Eu060-3 | Eu06013 |
| [x]oth unspec antiepileptics caus adverse eff in therap use | Epilepsy | Epilepsy or seizures | U6066 | U606600 |
| [x]other epilepsy | Epilepsy | Epilepsy or seizures | Fyu51 | Fyu5100 |
| [x]other generalized epilepsy and epileptic syndromes | Epilepsy | Epilepsy or seizures | Fyu50 | Fyu5000 |
| [x]other status epilepticus | Epilepsy | Epilepsy or seizures | Fyu52 | Fyu5200 |
| [x]status epilepticus, unspecified | Epilepsy | Epilepsy or seizures | Fyu59 | Fyu5900 |
| 1 to 12 seizures a year | Epilepsy | Epilepsy or seizures | 667Q | 667Q.00 |
| 1 to 7 seizures a week | Epilepsy | Epilepsy or seizures | 667S | 667S.00 |
| 2 to 4 seizures a month | Epilepsy | Epilepsy or seizures | 667R | 667R.00 |
| absence seizure | Epilepsy | Epilepsy or seizures | 2828 | 2828 |
| adverse reaction to antiepileptics | Epilepsy | Epilepsy or seizures | ALLERGY4.8 |  |
| adverse reaction to drugs used in control of epilepsy | Epilepsy | Epilepsy or seizures | ALLERGY4.8.1 |  |
| benign rolandic epilepsy | Epilepsy | Epilepsy or seizures | F25y4 | F25y400 |
| complex partial epileptic seizure | Epilepsy | Epilepsy or seizures | F2545 | F254500 |
| complex partial status epilepticus | Epilepsy | Epilepsy or seizures | F25y3 | F25y300 |
| contraceptiv advice for patients with epilepsy not indicated | Epilepsy | Epilepsy or seizures | 8IB2 | 8IB2.00 |
| contraceptive advice for patients on valproate for epilepsy | Epilepsy | Epilepsy or seizures | EMISNQCO292 |  |
| contraceptive advice for patients with epilepsy declined | Epilepsy | Epilepsy or seizures | 8IAg | 8IAg.00 |
| daily seizures | Epilepsy | Epilepsy or seizures | 667T | 667T.00 |
| emergency epilepsy treatment since last appointment | Epilepsy | Epilepsy or seizures | 667W | 667W.00 |
| epilepsy | Epilepsy | Epilepsy or seizures | F25 | F25..00 |
| epilepsy associated problems | Epilepsy | Epilepsy or seizures | 6674 | 6674 |
| epilepsy associated problems | Epilepsy | Epilepsy or seizures | PC0678 |  |
| epilepsy care arrangement | Epilepsy | Epilepsy or seizures | 667E | 667E.00 |
| epilepsy confirmed | Epilepsy | Epilepsy or seizures | 1O30 | 1O30.00 |
| epilepsy control good | Epilepsy | Epilepsy or seizures | 667C | 667C.00 |
| epilepsy control poor | Epilepsy | Epilepsy or seizures | 667D | 667D.00 |
| epilepsy drug side effects | Epilepsy | Epilepsy or seizures | 6677 | 6677 |
| epilepsy drug side effects | Epilepsy | Epilepsy or seizures | PC0681 |  |
| epilepsy limits activities | Epilepsy | Epilepsy or seizures | 667K | 667K.00 |
| epilepsy management plan given | Epilepsy | Epilepsy or seizures | 667M | 667M.00 |
| epilepsy medication review | Epilepsy | Epilepsy or seizures | 8BIF | 8BIF.00 |
| epilepsy monitoring | Epilepsy | Epilepsy or seizures | 667 | 667..00 |
| epilepsy monitoring | Epilepsy | Epilepsy or seizures | PC0037 |  |
| epilepsy monitoring administration | Epilepsy | Epilepsy or seizures | EMISNQEP19 |  |
| epilepsy monitoring call first letter | Epilepsy | Epilepsy or seizures | 9Of5 | 9Of5.00 |
| epilepsy monitoring call first letter | Epilepsy | Epilepsy or seizures | EMISNQEP5 |  |
| epilepsy monitoring call second letter | Epilepsy | Epilepsy or seizures | 9Of6 | 9Of6.00 |
| epilepsy monitoring call second letter | Epilepsy | Epilepsy or seizures | EMISNQEP6 |  |
| epilepsy monitoring in primary care | Epilepsy | Epilepsy or seizures | EMISNQEP20 |  |
| epilepsy monitoring in secondary care | Epilepsy | Epilepsy or seizures | EMISNQEP21 |  |
| epilepsy monitoring nos | Epilepsy | Epilepsy or seizures | 667Z | 667Z.00 |
| epilepsy monitoring telephone invite | Epilepsy | Epilepsy or seizures | 9Of4 | 9Of4.00 |
| epilepsy monitoring verbal invite | Epilepsy | Epilepsy or seizures | 9Of3 | 9Of3.00 |
| epilepsy nos | Epilepsy | Epilepsy or seizures | F25z | F25z.00 |
| epilepsy prevents employment | Epilepsy | Epilepsy or seizures | 667H | 667H.00 |
| epilepsy restricts employment | Epilepsy | Epilepsy or seizures | 667G | 667G.00 |
| epilepsy severity | Epilepsy | Epilepsy or seizures | 667N | 667N.00 |
| epilepsy treatment changed | Epilepsy | Epilepsy or seizures | 6678 | 6678 |
| epilepsy treatment compliance good | Epilepsy | Epilepsy or seizures | EMISNQEP23 |  |
| epilepsy treatment compliance poor | Epilepsy | Epilepsy or seizures | EMISNQEP24 |  |
| epilepsy treatment continued | Epilepsy | Epilepsy or seizures | EMISNQEP4 |  |
| epilepsy treatment started | Epilepsy | Epilepsy or seizures | 6679 | 6679 |
| epilepsy treatment stopped | Epilepsy | Epilepsy or seizures | 667A | 667A.00 |
| epileptic absences | Epilepsy | Epilepsy or seizures | F2500-1 | F250011 |
| epileptic automatism | Epilepsy | Epilepsy or seizures | F2544 | F254400 |
| epileptic seizures - akinetic | Epilepsy | Epilepsy or seizures | F2503 | F250300 |
| epileptic seizures - atonic | Epilepsy | Epilepsy or seizures | F2502 | F250200 |
| epileptic seizures - clonic | Epilepsy | Epilepsy or seizures | F2512 | F251200 |
| epileptic seizures - myoclonic | Epilepsy | Epilepsy or seizures | F2513 | F251300 |
| epileptic seizures - tonic | Epilepsy | Epilepsy or seizures | F2514 | F251400 |
| exception reporting: epilepsy quality indicators | Epilepsy | Epilepsy or seizures | 9h6 | 9h6..00 |
| fit (in known epileptic) nos | Epilepsy | Epilepsy or seizures | F25z-1 | F25z.11 |
| focal epilepsy | Epilepsy | Epilepsy or seizures | F2550-1 | F255011 |
| follow-up epilepsy assessment | Epilepsy | Epilepsy or seizures | 6672 | 6672 |
| follow-up epilepsy assessment | Epilepsy | Epilepsy or seizures | PC0677 |  |
| gelastic epilepsy | Epilepsy | Epilepsy or seizures | F25y1 | F25y100 |
| generalised convulsive epilepsy | Epilepsy | Epilepsy or seizures | F251 | F251.00 |
| generalised convulsive epilepsy nos | Epilepsy | Epilepsy or seizures | F251z | F251z00 |
| generalised nonconvulsive epilepsy | Epilepsy | Epilepsy or seizures | F250 | F250.00 |
| generalised nonconvulsive epilepsy nos | Epilepsy | Epilepsy or seizures | F250z | F250z00 |
| generalised seizure | Epilepsy | Epilepsy or seizures | F25H | F25H.00 |
| grand mal (major) epilepsy | Epilepsy | Epilepsy or seizures | F2510 | F251000 |
| grand mal seizure | Epilepsy | Epilepsy or seizures | F2516 | F251600 |
| grand mal status | Epilepsy | Epilepsy or seizures | F253 | F253.00 |
| h/o: epilepsy | Epilepsy | Epilepsy or seizures | 1473 | 1473 |
| h/o: seizures | Epilepsy | Epilepsy or seizures | JHCHO1 |  |
| honos-ld item 13 - seizures | Epilepsy | Epilepsy or seizures | EMISNQHO38 |  |
| hypsarrhythmia | Epilepsy | Epilepsy or seizures | F2560 | F256000 |
| infantile spasms | Epilepsy | Epilepsy or seizures | F256 | F256.00 |
| infantile spasms nos | Epilepsy | Epilepsy or seizures | F256z | F256z00 |
| initial epilepsy assessment | Epilepsy | Epilepsy or seizures | PC0676 |  |
| initial epilepsy assessment | Epilepsy | Epilepsy or seizures | 6671 | 6671 |
| jacksonian, focal or motor epilepsy | Epilepsy | Epilepsy or seizures | F2550 | F255000 |
| jacksonian, focal or motor epilepsy | Epilepsy | Epilepsy or seizures | F2550 | F255000 |
| juvenile absence epilepsy | Epilepsy | Epilepsy or seizures | F2504 | F250400 |
| juvenile myoclonic epilepsy | Epilepsy | Epilepsy or seizures | F25A | F25A.00 |
| kojevnikovs epilepsy | Epilepsy | Epilepsy or seizures | F257 | F257.00 |
| lennox-gastaut syndrome | Epilepsy | Epilepsy or seizures | F2505 | F250500 |
| lightning spasms | Epilepsy | Epilepsy or seizures | F256-1 | F256.11 |
| limbic system epilepsy | Epilepsy | Epilepsy or seizures | F2543 | F254300 |
| locl-rlt(foc)(part)idiop epilep&epilptic syn seiz locl onset | Epilepsy | Epilepsy or seizures | F25y2 | F25y200 |
| many seizures a day | Epilepsy | Epilepsy or seizures | 667V | 667V.00 |
| menstrual epilepsy | Epilepsy | Epilepsy or seizures | F25D | F25D.00 |
| motor epilepsy | Epilepsy | Epilepsy or seizures | F2550-2 | F255012 |
| myoclonic seizure | Epilepsy | Epilepsy or seizures | F132z-2 | F132z12 |
| neonatal myoclonic epilepsy | Epilepsy | Epilepsy or seizures | F2511 | F251100 |
| nocturnal epilepsy | Epilepsy | Epilepsy or seizures | PC0682 |  |
| nocturnal epilepsy | Epilepsy | Epilepsy or seizures | 667B | 667B.00 |
| ohtahara syndrome | Epilepsy | Epilepsy or seizures | F259-1 | F259.11 |
| other forms of epilepsy | Epilepsy | Epilepsy or seizures | F25y | F25y.00 |
| other forms of epilepsy nos | Epilepsy | Epilepsy or seizures | F25yz | F25yz00 |
| other specified generalised convulsive epilepsy | Epilepsy | Epilepsy or seizures | F251y | F251y00 |
| other specified generalised nonconvulsive epilepsy | Epilepsy | Epilepsy or seizures | F250y | F250y00 |
| otohara syndrome | Epilepsy | Epilepsy or seizures | F2511-1 | F251111 |
| panayiotopoulos syndrome | Epilepsy | Epilepsy or seizures | F25y5 | F25y500 |
| partial epilepsy | Epilepsy | Epilepsy or seizures | EMISR4QPA1 |  |
| partial epilepsy with autonomic symptoms | Epilepsy | Epilepsy or seizures | F2553-1 | F255311 |
| partial epilepsy with impairment of consciousness | Epilepsy | Epilepsy or seizures | F254 | F254.00 |
| partial epilepsy with impairment of consciousness nos | Epilepsy | Epilepsy or seizures | F254z | F254z00 |
| partial epilepsy without impairment of consciousness | Epilepsy | Epilepsy or seizures | F255 | F255.00 |
| partial epilepsy without impairment of consciousness nos | Epilepsy | Epilepsy or seizures | F255z | F255z00 |
| partial epilepsy without impairment of consciousness os | Epilepsy | Epilepsy or seizures | F255y | F255y00 |
| petit mal (minor) epilepsy | Epilepsy | Epilepsy or seizures | F2500 | F250000 |
| petit mal status | Epilepsy | Epilepsy or seizures | F252 | F252.00 |
| photosensitive epilepsy | Epilepsy | Epilepsy or seizures | F25F | F25F.00 |
| post-ictal state | Epilepsy | Epilepsy or seizures | F258 | F258.00 |
| pre-conception advice for patients with epilepsy | Epilepsy | Epilepsy or seizures | 67IJ0 | 67IJ000 |
| pre-conception advice for patients with epilepsy declined | Epilepsy | Epilepsy or seizures | 8IAh | 8IAh.00 |
| pregnancy advice for patients with epilepsy | Epilepsy | Epilepsy or seizures | 67AF | 67AF.00 |
| pregnancy advice for patients with epilepsy declined | Epilepsy | Epilepsy or seizures | 8IAi | 8IAi.00 |
| progressive myoclonic epilepsy | Epilepsy | Epilepsy or seizures | F1321 | F132100 |
| psychomotor epilepsy | Epilepsy | Epilepsy or seizures | F2541 | F254100 |
| psychosensory epilepsy | Epilepsy | Epilepsy or seizures | F2542 | F254200 |
| pykno-epilepsy | Epilepsy | Epilepsy or seizures | F2501 | F250100 |
| reason for referral: epilepsy | Epilepsy | Epilepsy or seizures | EMISNQRE225 |  |
| salaam attacks | Epilepsy | Epilepsy or seizures | F2561 | F256100 |
| seizure free >12 months | Epilepsy | Epilepsy or seizures | 667F | 667F.00 |
| sensory induced epilepsy | Epilepsy | Epilepsy or seizures | F2551 | F255100 |
| severe myoclonic epilepsy in infancy | Epilepsy | Epilepsy or seizures | F25G | F25G.00 |
| simple partial epileptic seizure | Epilepsy | Epilepsy or seizures | F2556 | F255600 |
| somatosensory epilepsy | Epilepsy | Epilepsy or seizures | F2552 | F255200 |
| status epilepticus | Epilepsy | Epilepsy or seizures | F253-1 | F253.11 |
| status epilepticus, unspecified | Epilepsy | Epilepsy or seizures | F25X | F25X.00 |
| status epilepticus, unspecified | Epilepsy | Epilepsy or seizures | F25X | F25X.00 |
| stress-induced epilepsy | Epilepsy | Epilepsy or seizures | F25E | F25E.00 |
| sudep - sudden unexpected death in epilepsy | Epilepsy | Epilepsy or seizures | 94F1 |  |
| temporal lobe epilepsy | Epilepsy | Epilepsy or seizures | F2540 | F254000 |
| tonic-clonic epilepsy | Epilepsy | Epilepsy or seizures | F2515 | F251500 |
| tonic-clonic epilepsy | Epilepsy | Epilepsy or seizures | F2510-1 | F251011 |
| transient epileptic amnesia | Epilepsy | Epilepsy or seizures | 1B1W | 1B1W.00 |
| traumatic epilepsy | Epilepsy | Epilepsy or seizures | SC200 | SC20000 |
| tremors/seizures | Epilepsy | Epilepsy or seizures | EMISCTR3 |  |
| unilateral epilepsy | Epilepsy | Epilepsy or seizures | F2555 | F255500 |
| visceral reflex epilepsy | Epilepsy | Epilepsy or seizures | F2553 | F255300 |
| visual reflex epilepsy | Epilepsy | Epilepsy or seizures | F2554 | F255400 |
| west syndrome | Epilepsy | Epilepsy or seizures | F256-2 | F256.12 |
| adult spinal muscular atrophy | Spinal muscular atrophy | Motor neuron diseases or related disorders | F1512 | F151200 |
| amyotrophic lateral sclerosis | MND | Motor neuron diseases or related disorders | F1520 | F152000 |
| amyotrophic lateral sclerosis drugs band 1 | MND | Motor neuron diseases or related disorders | 7Q041 | 7Q04100 |
| amyotrophic lateral sclerosis functional rating scale - rvsd | MND | Motor neuron diseases or related disorders | 38VT |  |
| anterior horn cell disease | MND | Motor neuron diseases or related disorders | F15 | F15..00 |
| anterior horn cell disease nos | MND | Motor neuron diseases or related disorders | F15z | F15z.00 |
| infantile spinal muscular atrophy | Spinal muscular atrophy | Motor neuron diseases or related disorders | F150-1 | F150.11 |
| juvenile spinal muscular atrophy | Spinal muscular atrophy | Motor neuron diseases or related disorders | F1511-1 | F151111 |
| progressive bulbar palsy | MND | Motor neuron diseases or related disorders | F1522 | F152200 |
| progressive muscular atrophy | MND | Motor neuron diseases or related disorders | F1521 | F152100 |
| pseudobulbar palsy | MND | Motor neuron diseases or related disorders | F1523 | F152300 |
| spinal muscular atrophy | Spinal muscular atrophy | Motor neuron diseases or related disorders | F151 | F151.00 |
| spinal muscular atrophy nos | Spinal muscular atrophy | Motor neuron diseases or related disorders | F151z | F151z00 |
| unspecified spinal muscular atrophy | Spinal muscular atrophy | Motor neuron diseases or related disorders | F1510 | F151000 |
| [x]other chorea | Huntington/Chorea | Movement disorders other | Fyu26 | Fyu2600 |
| drug-induced chorea | Chorea | Movement disorders other | F1352 | F135200 |
| extrapyramidal disease excluding parkinsons disease | Movement disorders | Movement disorders other | F13-1 | F13..11 |
| hemiballismus | Chorea | Movement disorders other | F1350 | F135000 |
| huntingtons chorea | Huntington/Chorea | Movement disorders other | F134 | F134.00 |
| other choreas | Huntington/Chorea | Movement disorders other | F135 | F135.00 |
| other choreas nos | Huntington/Chorea | Movement disorders other | F135z | F135z00 |
| paroxysmal choreo-athetosis | Dystonic disorders | Movement disorders other | F1351 | F135100 |
| rheumatic chorea | Huntington/Chorea | Movement disorders other | G02 | G02..00 |
| rheumatic chorea nos | Huntington/Chorea | Movement disorders other | G02z | G02z.00 |
| rheumatic chorea with heart involvement | Huntington/Chorea | Movement disorders other | G020 | G020.00 |
| rheumatic chorea without mention of heart involvement | Huntington/Chorea | Movement disorders other | G021 | G021.00 |
| sydenhams chorea | Huntington/Chorea | Movement disorders other | G02-1 | G02..11 |
| [rfc] multiple sclerosis | Multiple sclerosis or other white matter disorders | Multiple sclerosis or other white matter disorders | HNG0052 |  |
| [rfc] multiple sclerosis | Multiple sclerosis or other white matter disorders | Multiple sclerosis or other white matter disorders | HNG0603 |  |
| [x]acute disseminated demyelination, unspecified | Multiple sclerosis or other white matter disorders | Multiple sclerosis or other white matter disorders | Fyu42 | Fyu4200 |
| [x]demyelinating diseases of the central nervous system | Multiple sclerosis or other white matter disorders | Multiple sclerosis or other white matter disorders | Fyu4 | Fyu4.00 |
| [x]other specified acute disseminated demyelination | Multiple sclerosis or other white matter disorders | Multiple sclerosis or other white matter disorders | Fyu40 | Fyu4000 |
| [x]other specified demyelinating diseases/the cns | Multiple sclerosis or other white matter disorders | Multiple sclerosis or other white matter disorders | Fyu41 | Fyu4100 |
| acute disseminated demyelination, unspecified | Multiple sclerosis or other white matter disorders | Multiple sclerosis or other white matter disorders | F21X | F21X.00 |
| benign multiple sclerosis | Multiple sclerosis or other white matter disorders | Multiple sclerosis or other white matter disorders | F204 | F204.00 |
| central demyelination of corpus callosum | Multiple sclerosis or other white matter disorders | Multiple sclerosis or other white matter disorders | F21y3 | F21y300 |
| central nervous system demyelination nos | Multiple sclerosis or other white matter disorders | Multiple sclerosis or other white matter disorders | F21z | F21z.00 |
| exacerbation of multiple sclerosis | Multiple sclerosis or other white matter disorders | Multiple sclerosis or other white matter disorders | F203 | F203.00 |
| generalised multiple sclerosis | Multiple sclerosis or other white matter disorders | Multiple sclerosis or other white matter disorders | F202 | F202.00 |
| management of multiple sclerosis in early disease phase | Multiple sclerosis or other white matter disorders | Multiple sclerosis or other white matter disorders | 8Cc1 | 8Cc1.00 |
| management of multiple sclerosis in onset phase | Multiple sclerosis or other white matter disorders | Multiple sclerosis or other white matter disorders | 8Cc0 | 8Cc0.00 |
| management of multiple sclerosis in palliative phase | Multiple sclerosis or other white matter disorders | Multiple sclerosis or other white matter disorders | 8Cc4 | 8Cc4.00 |
| management of multiple sclerosis in stable disability phase | Multiple sclerosis or other white matter disorders | Multiple sclerosis or other white matter disorders | 8Cc2 | 8Cc2.00 |
| multiple sclerosis | Multiple sclerosis or other white matter disorders | Multiple sclerosis or other white matter disorders | F20 | F20..00 |
| multiple sclerosis - personal health plan | Multiple sclerosis or other white matter disorders | Multiple sclerosis or other white matter disorders | EMISNQMU8 |  |
| multiple sclerosis - primary progressive | Multiple sclerosis or other white matter disorders | Multiple sclerosis or other white matter disorders | EMISNQMU11 |  |
| multiple sclerosis - relapsing remitting | Multiple sclerosis or other white matter disorders | Multiple sclerosis or other white matter disorders | EMISNQMU13 |  |
| multiple sclerosis - secondary progressive | Multiple sclerosis or other white matter disorders | Multiple sclerosis or other white matter disorders | EMISNQMU12 |  |
| multiple sclerosis care plan agreed | Multiple sclerosis or other white matter disorders | Multiple sclerosis or other white matter disorders | 8CS1 | 8CS1.00 |
| multiple sclerosis monitoring first letter | Multiple sclerosis or other white matter disorders | Multiple sclerosis or other white matter disorders | EMISNQMU21 |  |
| multiple sclerosis monitoring first letter | Multiple sclerosis or other white matter disorders | Multiple sclerosis or other white matter disorders | 9mD0 | 9mD0.00 |
| multiple sclerosis multidisciplinary review | Multiple sclerosis or other white matter disorders | Multiple sclerosis or other white matter disorders | 666B | 666B.00 |
| multiple sclerosis nos | Multiple sclerosis or other white matter disorders | Multiple sclerosis or other white matter disorders | F20z | F20z.00 |
| multiple sclerosis of the brain stem | Multiple sclerosis or other white matter disorders | Multiple sclerosis or other white matter disorders | F200 | F200.00 |
| multiple sclerosis of the spinal cord | Multiple sclerosis or other white matter disorders | Multiple sclerosis or other white matter disorders | F201 | F201.00 |
| multiple sclerosis review | Multiple sclerosis or other white matter disorders | Multiple sclerosis or other white matter disorders | 666A | 666A.00 |
| neuromyelitis optica | Multiple sclerosis or other white matter disorders | Multiple sclerosis or other white matter disorders | F210 | F210.00 |
| other central nervous system demyelinating diseases | Multiple sclerosis or other white matter disorders | Multiple sclerosis or other white matter disorders | F21 | F21..00 |
| other specified central nervous system demyelinating disease | Multiple sclerosis or other white matter disorders | Multiple sclerosis or other white matter disorders | F21y | F21y.00 |
| other specified central nervous system demyelination nos | Multiple sclerosis or other white matter disorders | Multiple sclerosis or other white matter disorders | F21yz | F21yz00 |
| primary progressive multiple sclerosis | Multiple sclerosis or other white matter disorders | Multiple sclerosis or other white matter disorders | F206 | F206.00 |
| progressive multifocal leucoencephalopathy | Multiple sclerosis or other white matter disorders | Multiple sclerosis or other white matter disorders | A413 | A413.00 |
| progressive multifocal leukoencephalopathy | Multiple sclerosis or other white matter disorders | Multiple sclerosis or other white matter disorders | A413-1 | A413.11 |
| referral by multiple sclerosis nurse specialist | Multiple sclerosis or other white matter disorders | Multiple sclerosis or other white matter disorders | EMISNQRE383 |  |
| referral to community multiple sclerosis team | Multiple sclerosis or other white matter disorders | Multiple sclerosis or other white matter disorders | 8Hkv | 8Hkv.00 |
| relapsing and remitting multiple sclerosis | Multiple sclerosis or other white matter disorders | Multiple sclerosis or other white matter disorders | F207 | F207.00 |
| schilders disease | Multiple sclerosis or other white matter disorders | Multiple sclerosis or other white matter disorders | F211 | F211.00 |
| secondary progressive multiple sclerosis | Multiple sclerosis or other white matter disorders | Multiple sclerosis or other white matter disorders | F208 | F208.00 |
| spec serv for pat with multiple sclerosis - enh serv admin | Multiple sclerosis or other white matter disorders | Multiple sclerosis or other white matter disorders | 9kG | 9kG..00 |
| spinal demyelination | Multiple sclerosis or other white matter disorders | Multiple sclerosis or other white matter disorders | EMISR4QSP2 |  |
| transverse myelitis | Multiple sclerosis or other white matter disorders | Multiple sclerosis or other white matter disorders | F03-3 | F03..13 |
| transverse myelitis | Multiple sclerosis or other white matter disorders | Multiple sclerosis or other white matter disorders | F037 | F037.00 |
| varicella transverse myelitis | Multiple sclerosis or other white matter disorders | Multiple sclerosis or other white matter disorders | F0370 | F037000 |
| [d]transient monoplegia nos | Paralytic symptoms | Paralysis | R0140 | R014000 |
| [d]transient paralysis of a limb | Paralytic symptoms | Paralysis | R014 | R014.00 |
| acute flaccid paralysis | Paralytic symptoms | Paralysis | JHCAC3 |  |
| diplegia of upper limbs | Paralytic symptoms | Paralysis | F242 | F242.00 |
| exercises for paralysis | Paralytic symptoms | Paralysis | 8E53 | 8E53.00 |
| familial hypokalaemic periodic paralysis | Paralytic symptoms | Paralysis | F393-1 | F393.11 |
| familial periodic paralysis | Paralytic symptoms | Paralysis | F393 | F393.00 |
| flaccid hemiplegia | Paralytic symptoms | Paralysis | F220 | F220.00 |
| flaccid paraplegia | Paralytic symptoms | Paralysis | F2410 | F241000 |
| flaccid tetraplegia | Paralytic symptoms | Paralysis | F2400 | F240000 |
| general paralysis of insane | Paralytic symptoms | Paralysis | A941-1 | A941.11 |
| hemiplegia | Paralytic symptoms | Paralysis | F22 | F22..00 |
| hemiplegia nos | Paralytic symptoms | Paralysis | F22z | F22z.00 |
| hysterical paralysis | Paralytic symptoms | Paralysis | E2014 | E201400 |
| infantile hemiplegia nos | Paralytic symptoms | Paralysis | F234 | F234.00 |
| left hemiplegia | Paralytic symptoms | Paralysis | EGTONLE2 |  |
| left hemiplegia | Paralytic symptoms | Paralysis | F222 | F222.00 |
| massive muscular calcification associated with paraplegia | Paralytic symptoms | Paralysis | N2310-1 | N231011 |
| monoplegia of lower limb | Paralytic symptoms | Paralysis | F243 | F243.00 |
| monoplegia of upper limb | Paralytic symptoms | Paralysis | F244 | F244.00 |
| monoplegia unspecified | Paralytic symptoms | Paralysis | F245 | F245.00 |
| o/e - diplegia | Paralytic symptoms | Paralysis | 2837 | 2837 |
| o/e - hemiplegia | Paralytic symptoms | Paralysis | 2833 | 2833 |
| o/e - monoplegia | Paralytic symptoms | Paralysis | 2834 | 2834 |
| o/e - paralysis | Paralytic symptoms | Paralysis | 283 | 283..00 |
| o/e - paralysis nos | Paralytic symptoms | Paralysis | 283Z | 283Z.00 |
| o/e - paraplegia | Paralytic symptoms | Paralysis | 2835 | 2835 |
| o/e - quadriplegia | Paralytic symptoms | Paralysis | 2836 | 2836 |
| paralysis following electric shock | Paralytic symptoms | Paralysis | SN481-1 | SN48111 |
| paralysis following electric shock | Paralytic symptoms | Paralysis | SN481-2 | SN48112 |
| paralysis nos | Paralytic symptoms | Paralysis | F24z | F24z.00 |
| paralysis present | Paralytic symptoms | Paralysis | 1B33 | 1B33.00 |
| paraplegia | Paralytic symptoms | Paralysis | F241 | F241.00 |
| paraplegia - congenital | Paralytic symptoms | Paralysis | F230-1 | F230.11 |
| psychogenic paralysis | Paralytic symptoms | Paralysis | E2600 | E260000 |
| quadriplegia | Paralytic symptoms | Paralysis | F240 | F240.00 |
| right hemiplegia | Paralytic symptoms | Paralysis | EGTONRI2 |  |
| right hemiplegia | Paralytic symptoms | Paralysis | F223 | F223.00 |
| spastic hemiplegia | Paralytic symptoms | Paralysis | F221 | F221.00 |
| spastic tetraplegia | Paralytic symptoms | Paralysis | F2401 | F240100 |
| supranuclear paralysis | Paralytic symptoms | Paralysis | F36y0 | F36y000 |
| tetraplegia | Paralytic symptoms | Paralysis | F240-1 | F240.11 |
| tetraplegia - congenital | Paralytic symptoms | Paralysis | F232-1 | F232.11 |
| upper limb flaccidity | Paralytic symptoms | Paralysis | 294E | 294E.00 |
| [rfc] parkinsons disease | Parkinson's Disease | Parkinson's Disease | HNG0633 |  |
| [rfc] parkinsons disease | Parkinson's Disease | Parkinson's Disease | HNG0054 |  |
| [x]dementia in parkinsons disease | Parkinson's Disease | Parkinson's Disease | Eu023 | Eu02300 |
| history of parkinsons disease | Parkinson's Disease | Parkinson's Disease | 147F | 147F.00 |
| lindop parkinsons assessment scale - bed mobility score | Parkinson's Disease | Parkinson's Disease | EMISNQLI69 |  |
| lindop parkinsons assessment scale - gait mobility score | Parkinson's Disease | Parkinson's Disease | EMISNQLI62 |  |
| o/e-festination-parkinson gait | Parkinson's Disease | Parkinson's Disease | 2994 | 2994 |
| o/e - parkinson posture | Parkinson's Disease | Parkinson's Disease | 2987-1 | 2987.11 |
| o/e - parkinsonian tremor | Parkinson's Disease | Parkinson's Disease | 297A | 297A.00 |
| o/e -parkinson flexion posture | Parkinson's Disease | Parkinson's Disease | 2987 | 2987 |
| paralysis agitans | Parkinson's Disease | Parkinson's Disease | F120 | F120.00 |
| parkinsons disease | Parkinson's Disease | Parkinson's Disease | F12 | F12..00 |
| parkinsons disease nos | Parkinson's Disease | Parkinson's Disease | F12z | F12z.00 |
| progressive supranuclear palsy | Parkinson's Disease | Parkinson's Disease | F24y0 | F24y000 |
| reason for referral: parkinsons disease | Parkinson's Disease | Parkinson's Disease | EMISNQRE248 |  |
| referral by parkinsons disease nurse specialist | Parkinson's Disease | Parkinson's Disease | EMISNQRE380 |  |
| seen by parkinsons service | Parkinson's Disease | Parkinson's Disease | EMISNQSE120 |  |
| [x]other drug-induced secondary parkinsonism | Parkinson's Disease | Parkinsonism other | Fyu20 | Fyu2000 |
| [x]other secondary parkinsonism | Parkinson's Disease | Parkinsonism other | Fyu21 | Fyu2100 |
| [x]parkinsonism in diseases classified elsewhere | Parkinson's Disease | Parkinsonism other | Fyu22 | Fyu2200 |
| [x]secondary parkinsonism, unspecified | Parkinson's Disease | Parkinsonism other | Fyu29 | Fyu2900 |
| drug induced parkinsonism | Parkinson's Disease | Parkinsonism other | F121-1 | F121.11 |
| parkinsonism secondary to drugs | Parkinson's Disease | Parkinsonism other | F121 | F121.00 |
| parkinsonism with orthostatic hypotension | Parkinson's Disease | Parkinsonism other | F1303 | F130300 |
| postencephalitic parkinsonism | Parkinson's Disease | Parkinsonism other | F123 | F123.00 |
| secondary parkinsonism, unspecified | Parkinson's Disease | Parkinsonism other | F12X | F12X.00 |
| secondary parkinsonism, unspecified | Parkinson's Disease | Parkinsonism other | F12X | F12X.00 |
| syphilitic parkinsonism | Parkinson's Disease | Parkinsonism other | A94y1 | A94y100 |
| vascular parkinsonism | Parkinson's Disease | Parkinsonism other | F124 | F124.00 |
| [d]spastic gait | Spasticity | Spinal cord disorders | R0122 | R012200 |
| cauda equina injury without bony injury | Cauda equina syndrome | Spinal cord disorders | SJ24 | SJ24.00 |
| cauda equina syndrome | Cauda equina syndrome | Spinal cord disorders | F246 | F246.00 |
| cauda equina syndrome nos | Cauda equina syndrome | Spinal cord disorders | F246z | F246z00 |
| cauda equina syndrome not affecting bladder | Cauda equina syndrome | Spinal cord disorders | F2460 | F246000 |
| cauda equina syndrome with cord bladder | Cauda equina syndrome | Spinal cord disorders | F2461 | F246100 |
| closed injury cauda equina | Cauda equina syndrome | Spinal cord disorders | SJ240 | SJ24000 |
| exercises for spasticity | Spasticity | Spinal cord disorders | 8E54 | 8E54.00 |
| hereditary spastic paraplegia | Spinal cord disorders | Spinal cord disorders | F141 | F141.00 |
| lower limb spasticity | Spasticity | Spinal cord disorders | 2949 | 2949 |
| lumbar disc prolapse with cauda equina compression | Cauda equina syndrome | Spinal cord disorders | N12C3 | N12C300 |
| o/e - gait spastic | Spasticity | Spinal cord disorders | 2992 | 2992 |
| o/e - spastic gait | Spasticity | Spinal cord disorders | 2992-1 | 2992.11 |
| spastic foot | Spasticity | Spinal cord disorders | F221-1 | F221.11 |
| spastic paraparesis | Degenerative myelopathic disorders | Spinal cord disorders | EMISR4QSP1 |  |
| spastic paraplegia | Degenerative myelopathic disorders | Spinal cord disorders | F2411 | F241100 |
| tropical spastic paraplegia | Degenerative myelopathic disorders | Spinal cord disorders | F038 | F038.00 |
| upper limb spasticity | Spasticity | Spinal cord disorders | 294A | 294A.00 |
| worsening limb spasticity | Spasticity | Spinal cord disorders | 294B | 294B.00 |
| [x]other congenital hydrocephalus | Hydrocephalus | Structural developmental anomalies or disorders of CSF fluid pressure | Pyu01 | Pyu0100 |
| [x]other hydrocephalus | Hydrocephalus | Structural developmental anomalies or disorders of CSF fluid pressure | FyuA1 | FyuA100 |
| [x]post-traumatic hydrocephalus, unspecified | Hydrocephalus | Structural developmental anomalies or disorders of CSF fluid pressure | FyuAG | FyuAG00 |
| [x]unspecified spina bifida with hydrocephalus | Hydrocephalus | Structural developmental anomalies or disorders of CSF fluid pressure | Pyu04 | Pyu0400 |
| acquired communicating hydrocephalus | Hydrocephalus | Structural developmental anomalies or disorders of CSF fluid pressure | F113 | F113.00 |
| acquired hydrocephalus of newborn | Hydrocephalus | Structural developmental anomalies or disorders of CSF fluid pressure | Q48H |  |
| acquired obstructive hydrocephalus | Hydrocephalus | Structural developmental anomalies or disorders of CSF fluid pressure | F114 | F114.00 |
| cause of learning disabilities: congenital hydrocephalus | Hydrocephalus | Structural developmental anomalies or disorders of CSF fluid pressure | EMISNQCA49 |  |
| communicating hydrocephalus - acquired nos | Hydrocephalus | Structural developmental anomalies or disorders of CSF fluid pressure | F113z | F113z00 |
| congenital hydrocephalus | Hydrocephalus | Structural developmental anomalies or disorders of CSF fluid pressure | P23 | P23..00 |
| congenital hydrocephalus nos | Hydrocephalus | Structural developmental anomalies or disorders of CSF fluid pressure | P23z | P23z.00 |
| fissured spine with hydrocephalus | Hydrocephalus | Structural developmental anomalies or disorders of CSF fluid pressure | P102-1 | P102.11 |
| hydrocephalus | Hydrocephalus | Structural developmental anomalies or disorders of CSF fluid pressure | F115 | F115.00 |
| hydrocephalus with atresia of foramina of magendie+luschka | Hydrocephalus | Structural developmental anomalies or disorders of CSF fluid pressure | P233-2 | P233.12 |
| low pressure hydrocephalus | Hydrocephalus | Structural developmental anomalies or disorders of CSF fluid pressure | F1130-1 | F113011 |
| lumbar spina bifida with hydrocephalus | Hydrocephalus | Structural developmental anomalies or disorders of CSF fluid pressure | P1003 | P100300 |
| lumbar spina bifida with hydrocephalus - closed | Hydrocephalus | Structural developmental anomalies or disorders of CSF fluid pressure | P1033 | P103300 |
| lumbar spina bifida with hydrocephalus - open | Hydrocephalus | Structural developmental anomalies or disorders of CSF fluid pressure | P1023 | P102300 |
| lumbar spina bifida without hydrocephalus - closed | Spina bifida | Structural developmental anomalies or disorders of CSF fluid pressure | P1183 | P118300 |
| lumbar spina bifida without hydrocephalus - open | Spina bifida | Structural developmental anomalies or disorders of CSF fluid pressure | P1173 | P117300 |
| lumbar spina bifida without mention of hydrocephalus | Spina bifida | Structural developmental anomalies or disorders of CSF fluid pressure | P1103 | P110300 |
| myelocele with hydrocephalus | Hydrocephalus | Structural developmental anomalies or disorders of CSF fluid pressure | P102-3 | P102.13 |
| normal pressure hydrocephalus | Hydrocephalus | Structural developmental anomalies or disorders of CSF fluid pressure | F1130 | F113000 |
| other specified spina bifida with hydrocephalus | Hydrocephalus | Structural developmental anomalies or disorders of CSF fluid pressure | P10y | P10y.00 |
| other specified spina bifida without hydrocephalus | Spina bifida | Structural developmental anomalies or disorders of CSF fluid pressure | P11y | P11y.00 |
| post-traumatic hydrocephalus, unspecified | Hydrocephalus | Structural developmental anomalies or disorders of CSF fluid pressure | F11X | F11X.00 |
| post-traumatic hydrocephalus, unspecified | Hydrocephalus | Structural developmental anomalies or disorders of CSF fluid pressure | F11X | F11X.00 |
| rachischisis with hydrocephalus | Hydrocephalus | Structural developmental anomalies or disorders of CSF fluid pressure | P102-4 | P102.14 |
| sacral spina bifida with hydrocephalus - closed | Hydrocephalus | Structural developmental anomalies or disorders of CSF fluid pressure | P1034 | P103400 |
| sacral spina bifida with hydrocephalus - open | Hydrocephalus | Structural developmental anomalies or disorders of CSF fluid pressure | P1024 | P102400 |
| sacral spina bifida without hydrocephalus - closed | Spina bifida | Structural developmental anomalies or disorders of CSF fluid pressure | P1184 | P118400 |
| sacral spina bifida without hydrocephalus - open | Spina bifida | Structural developmental anomalies or disorders of CSF fluid pressure | P1174 | P117400 |
| spina bifida with hydrocephalus | Spina bifida | Structural developmental anomalies or disorders of CSF fluid pressure | P10 | P10..00 |
| spina bifida with hydrocephalus - closed | Hydrocephalus | Structural developmental anomalies or disorders of CSF fluid pressure | P103 | P103.00 |
| spina bifida with hydrocephalus - open | Hydrocephalus | Structural developmental anomalies or disorders of CSF fluid pressure | P102 | P102.00 |
| spina bifida with hydrocephalus - open nos | Hydrocephalus | Structural developmental anomalies or disorders of CSF fluid pressure | P102z | P102z00 |
| spina bifida with hydrocephalus nos | Hydrocephalus | Structural developmental anomalies or disorders of CSF fluid pressure | P100z | P100z00 |
| spina bifida with hydrocephalus nos | Hydrocephalus | Structural developmental anomalies or disorders of CSF fluid pressure | P10z | P10z.00 |
| spina bifida with hydrocephalus of late onset | Hydrocephalus | Structural developmental anomalies or disorders of CSF fluid pressure | P104 | P104.00 |
| spina bifida with hydrocephalus, unspecified | Hydrocephalus | Structural developmental anomalies or disorders of CSF fluid pressure | P1000 | P100000 |
| spina bifida without hydrocephalus - closed | Spina bifida | Structural developmental anomalies or disorders of CSF fluid pressure | P118 | P118.00 |
| spina bifida without hydrocephalus - closed nos | Spina bifida | Structural developmental anomalies or disorders of CSF fluid pressure | P118z | P118z00 |
| spina bifida without hydrocephalus - open | Spina bifida | Structural developmental anomalies or disorders of CSF fluid pressure | P117 | P117.00 |
| spina bifida without hydrocephalus - open nos | Spina bifida | Structural developmental anomalies or disorders of CSF fluid pressure | P117z | P117z00 |
| spina bifida without hydrocephalus, site unspecified | Spina bifida | Structural developmental anomalies or disorders of CSF fluid pressure | P1100 | P110000 |
| spina bifida without mention of hydrocephalus | Spina bifida | Structural developmental anomalies or disorders of CSF fluid pressure | P11 | P11..00 |
| spina bifida without mention of hydrocephalus nos | Spina bifida | Structural developmental anomalies or disorders of CSF fluid pressure | P11z | P11z.00 |
| thoracic spina bifida with hydrocephalus | Hydrocephalus | Structural developmental anomalies or disorders of CSF fluid pressure | P1002 | P100200 |
| thoracic spina bifida with hydrocephalus - open | Hydrocephalus | Structural developmental anomalies or disorders of CSF fluid pressure | P1022 | P102200 |
| thoracic spina bifida without hydrocephalus - open | Spina bifida | Structural developmental anomalies or disorders of CSF fluid pressure | P1172 | P117200 |
| thoracic spina bifida without mention of hydrocephalus | Spina bifida | Structural developmental anomalies or disorders of CSF fluid pressure | P1102 | P110200 |
| thoracolumbar spina bifida with hydrocephalus - closed | Hydrocephalus | Structural developmental anomalies or disorders of CSF fluid pressure | P103z-1 | P103z11 |
| unspecified spina bifida with hydrocephalus | Hydrocephalus | Structural developmental anomalies or disorders of CSF fluid pressure | P100 | P100.00 |
| unspecified spina bifida without hydrocephalus - closed | Spina bifida | Structural developmental anomalies or disorders of CSF fluid pressure | P1180 | P118000 |
| unspecified spina bifida without hydrocephalus nos | Spina bifida | Structural developmental anomalies or disorders of CSF fluid pressure | P110z | P110z00 |
| x-linked hydrocephalus | Hydrocephalus | Structural developmental anomalies or disorders of CSF fluid pressure | P235 | P235.00 |

### Supplementary Table 5: Relative prevalence of neurological conditions in people with SMI and SMI subtypes compared to the comparator population (unadjusted odds ratios)

|  | **Years before SMI diagnosis** | |  | **Index date** | **Years after SMI diagnosis** | | |
| --- | --- | --- | --- | --- | --- | --- | --- |
|  | -5 | -3 | -1 |  | 1 | 3 | 5 |
| **Multiple Sclerosis** |  |  |  |  |  |  |  |
| **All SMI** | 1.36 (1.13-1.62) | 1.36 (1.15-1.61) | 1.42 (1.21-1.66) | 1.44 (1.24-1.67) | 1.57 (1.36-1.83) | 1.51 (1.29-1.77) | 1.41 (1.17-1.70) |
| Schizophrenia | 0.61 (0.32-1.15) | 0.69 (0.39-1.22) | 0.71 (0.42-1.18) | 0.86 (0.54-1.39) | 1.03 (0.67-1.57) | 0.82 (0.51-1.34) | 0.69 (0.38-1.25) |
| Bipolar | 1.37 (1.04-1.81) | 1.41 (1.09-1.83) | 1.49 (1.17-1.89) | 1.47 (1.17-1.86) | 1.69 (1.34-2.12) | 1.60 (1.26-2.02) | 1.60 (1.22-2.10) |
| Other psychoses | 1.63 (1.26-2.12) | 1.55 (1.21-1.99) | 1.63 (1.29-2.06) | 1.62 (1.30-2.03) | 1.67 (1.34-2.09) | 1.69 (1.34-2.15) | 1.49 (1.11-2.00) |
| **Cerebrovascular disease** | |  |  |  |  |  |  |
| **All SMI** | 1.14 (1.06-1.22) | 1.16 (1.09-1.23) | 1.22 (1.15-1.28) | 1.33 (1.27-1.39) | 1.42 (1.36-1.48) | 1.43 (1.36-1.50) | 1.38 (1.30-1.46) |
| Schizophrenia | 0.90 (0.74-1.10) | 0.93 (0.79-1.11) | 0.95 (0.81-1.10) | 1.01 (0.88-1.16) | 1.19 (1.05-1.36) | 1.19 (1.04-1.36) | 1.21 (1.05-1.40) |
| Bipolar | 1.09 (0.94-1.25) | 1.15 (1.02-1.29) | 1.33 (1.21-1.47) | 1.46 (1.34-1.60) | 1.60 (1.47-1.74) | 1.66 (1.53-1.80) | 1.55 (1.42-1.71) |
| Other psychoses | 1.22 (1.12-1.34) | 1.22 (1.13-1.32) | 1.23 (1.15-1.32) | 1.35 (1.27-1.44) | 1.40 (1.32-1.48) | 1.38 (1.29-1.47) | 1.34 (1.23-1.45) |
| **Dementia** |  |  |  |  |  |  |  |
| **All SMI** | 1.12 (0.89-1.42) | 1.27 (1.07-1.51) | 1.70 (1.53-1.88) | 2.43 (2.25-2.64) | 3.70 (3.49-3.93) | 3.91 (3.67-4.16) | 3.21 (2.97-3.47) |
| Schizophrenia | 1.92 (1.10-3.33) | 1.63 (1.08-2.44) | 1.62 (1.22-2.16) | 2.23 (1.80-2.76) | 3.38 (2.85-4.01) | 3.61 (3.04-4.28) | 2.75 (2.25-3.37) |
| Bipolar | 1.90 (1.17-3.08) | 1.82 (1.21-2.73) | 2.03 (1.56-2.65) | 2.68 (2.22-3.24) | 3.62 (3.12-4.19) | 3.57 (3.10-4.12) | 2.96 (2.52-3.47) |
| Other psychoses | 0.84 (0.64-1.11) | 1.09 (0.91-1.31) | 1.64 (1.46-1.84) | 2.42 (2.21-2.65) | 3.82 (3.57-4.09) | 4.16 (3.86-4.50) | 3.54 (3.22-3.90) |
| **Ataxia** |  |  |  |  |  |  |  |
| **All SMI** | 2.12 (1.58-2.85) | 1.95 (1.48-2.56) | 1.82 (1.41-2.35) | 1.91 (1.50-2.43) | 2.27 (1.79-2.87) | 2.61 (2.04-3.34) | 2.54 (1.94-3.32) |
| Schizophrenia | 1.74 (0.82-3.69) | 1.73 (0.89-3.37) | 1.55 (0.83-2.91) | 1.58 (0.86-2.91) | 1.77 (1.03-3.03) | 1.50 (0.85-2.66) | 1.41 (0.74-2.71) |
| Bipolar | 1.64 (0.91-2.96) | 1.74 (1.03-2.95) | 1.65 (0.99-2.74) | 1.74 (1.06-2.84) | 2.21 (1.42-3.46) | 2.46 (1.58-3.81) | 2.71 (1.68-4.38) |
| Other psychoses | 2.53 (1.71-3.73) | 2.13 (1.49-3.06) | 1.98 (1.42-2.76) | 2.10 (1.53-2.88) | 2.46 (1.81-3.36) | 3.20 (2.32-4.41) | 3.02 (2.08-4.39) |
| **Epilepsy** |  |  |  |  |  |  |  |
| **All SMI** | 2.15 (2.03-2.28) | 2.21 (2.09-2.34) | 2.27 (2.16-2.39) | 2.40 (2.28-2.52) | 2.69 (2.57-2.83) | 2.85 (2.71-3.00) | 2.99 (2.81-3.17) |
| Schizophrenia | 2.01 (1.76-2.30) | 2.11 (1.85-2.40) | 2.11 (1.87-2.39) | 2.18 (1.95-2.45) | 2.66 (2.39-2.95) | 2.76 (2.47-3.08) | 3.00 (2.65-3.40) |
| Bipolar | 1.78 (1.60-1.97) | 1.85 (1.67-2.04) | 2.02 (1.84-2.22) | 2.17 (1.99-2.37) | 2.45 (2.25-2.66) | 2.62 (2.40-2.87) | 2.71 (2.45-3.01) |
| Other psychoses | 2.51 (2.32-2.72) | 2.54 (2.36-2.74) | 2.54 (2.36-2.72) | 2.66 (2.48-2.85) | 2.90 (2.71-3.10) | 3.09 (2.87-3.33) | 3.24 (2.97-3.55) |
| **Parkinson's disease** |  |  |  |  |  |  |  |
| **All SMI** | 3.06 (2.58-3.63) | 3.08 (2.64-3.58) | 2.77 (2.43-3.16) | 2.91 (2.58-3.29) | 3.18 (2.86-3.55) | 3.38 (3.03-3.78) | 3.34 (2.90-3.84) |
| Schizophrenia | 1.64 (1.01-2.66) | 1.53 (0.99-2.36) | 1.52 (1.03-2.24) | 2.00 (1.44-2.79) | 2.62 (1.99-3.45) | 3.68 (2.76-4.90) | 3.39 (2.44-4.72) |
| Bipolar | 1.33 (0.88-2.02) | 1.42 (1.00-2.02) | 1.68 (1.26-2.23) | 1.82 (1.40-2.36) | 2.25 (1.79-2.83) | 2.51 (1.98-3.18) | 2.58 (1.97-3.38) |
| Other psychoses | 4.49 (3.61-5.58) | 4.50 (3.70-5.47) | 3.63 (3.07-4.29) | 3.69 (3.17-4.30) | 3.81 (3.31-4.38) | 3.80 (3.28-4.40) | 3.89 (3.20-4.73) |
|  |  |  |  |  |  |  |  |
|  |  |  |  |  |  |  |  |
|  | **Years before SMI diagnosis** | |  | **Index date** | **Years after SMI diagnosis** | | |
|  | -5 | -3 | -1 |  | 1 | 3 | 5 |
| **Paralysis** |  |  |  |  |  |  |  |
| **All SMI** | 1.78 (1.48-2.15) | 1.78 (1.49-2.12) | 1.75 (1.49-2.07) | 1.79 (1.52-2.10) | 1.88 (1.60-2.20) | 1.94 (1.64-2.29) | 1.69 (1.40-2.05) |
| Schizophrenia | 1.18 (0.72-1.92) | 1.06 (0.66-1.71) | 1.15 (0.74-1.79) | 1.17 (0.76-1.80) | 1.25 (0.83-1.88) | 1.11 (0.72-1.71) | 1.10 (0.68-1.79) |
| Bipolar | 1.77 (1.30-2.42) | 1.82 (1.36-2.44) | 1.72 (1.30-2.28) | 1.77 (1.36-2.31) | 1.92 (1.48-2.48) | 2.03 (1.56-2.65) | 1.73 (1.27-2.36) |
| Other psychoses | 2.00 (1.57-2.55) | 2.01 (1.59-2.54) | 2.01 (1.60-2.52) | 2.04 (1.64-2.54) | 2.10 (1.69-2.60) | 2.26 (1.79-2.84) | 1.99 (1.50-2.63) |
| **Cerebral palsy** |  |  |  |  |  |  |  |
| **All SMI** | 2.55 (2.09-3.12) | 2.49 (2.05-3.04) | 2.47 (2.03-3.01) | 2.52 (2.08-3.05) | 2.63 (2.16-3.19) | 2.47 (2.00-3.04) | 2.42 (1.89-3.10) |
| Schizophrenia | 1.78 (1.10-2.87) | 1.79 (1.13-2.84) | 1.80 (1.15-2.83) | 1.87 (1.21-2.90) | 1.87 (1.21-2.90) | 1.67 (1.05-2.68) | 1.45 (0.81-2.58) |
| Bipolar | 2.80 (2.03-3.86) | 2.74 (1.99-3.77) | 2.66 (1.93-3.65) | 2.63 (1.91-3.61) | 2.84 (2.07-3.88) | 2.76 (1.95-3.91) | 2.31 (1.55-3.45) |
| Other psychoses | 2.74 (2.03-3.72) | 2.67 (1.97-3.60) | 2.69 (2.00-3.62) | 2.79 (2.09-3.73) | 2.87 (2.15-3.83) | 2.72 (1.99-3.71) | 3.30 (2.23-4.88) |
| **CSF disorders** |  |  |  |  |  |  |  |
| **All SMI** | 1.83 (1.33-2.51) | 1.79 (1.31-2.43) | 1.87 (1.39-2.51) | 2.01 (1.50-2.69) | 2.26 (1.68-3.04) | 2.28 (1.65-3.15) | 2.40 (1.70-3.38) |
| Schizophrenia | 1.55 (0.81-2.97) | 1.48 (0.80-2.77) | 1.51 (0.83-2.77) | 1.68 (0.95-2.99) | 1.95 (1.14-3.34) | 1.98 (1.15-3.42) | 2.09 (1.11-3.95) |
| Bipolar | 2.05 (1.22-3.42) | 1.92 (1.17-3.15) | 1.85 (1.14-2.99) | 1.92 (1.19-3.10) | 1.97 (1.23-3.14) | 2.00 (1.23-3.25) | 2.19 (1.27-3.78) |
| Other psychoses | 1.82 (1.11-3.00) | 1.86 (1.14-3.03) | 2.10 (1.34-3.29) | 2.28 (1.48-3.52) | 2.70 (1.78-4.11) | 2.79 (1.73-4.51) | 2.79 (1.65-4.72) |
| **Spinal cord disorders** | |  |  |  |  |  |  |
| **All SMI** | 1.30 (0.84-2.01) | 1.36 (0.91-2.03) | 1.44 (1.02-2.04) | 1.59 (1.15-2.19) | 1.69 (1.24-2.29) | 1.66 (1.19-2.31) | 1.42 (0.97-2.07) |
| Schizophrenia | 0.86 (0.25-2.98) | 0.71 (0.21-2.41) | 1.00 (0.37-2.66) | 1.20 (0.48-2.99) | 1.50 (0.70-3.23) | 1.19 (0.51-2.79) | 1.60 (0.66-3.86) |
| Bipolar | 2.26 (1.13-4.51) | 2.52 (1.36-4.66) | 2.11 (1.21-3.68) | 2.15 (1.27-3.66) | 2.17 (1.32-3.55) | 2.20 (1.32-3.68) | 1.71 (0.98-3.00) |
| Other psychoses | 0.90 (0.43-1.86) | 0.93 (0.48-1.81) | 1.16 (0.67-2.00) | 1.35 (0.84-2.18) | 1.43 (0.91-2.23) | 1.42 (0.89-2.28) | 1.09 (0.57-2.06) |
| **Disorders of nerve root, nerve plexus and peripheral nervous system** | | | | | | | |
| **All SMI** | 1.39 (0.97-1.99) | 1.27 (0.90-1.79) | 1.40 (1.01-1.93) | 1.52 (1.12-2.06) | 1.71 (1.27-2.30) | 1.91 (1.41-2.60) | 1.91 (1.38-2.66) |
| Schizophrenia | 1.48 (0.72-3.06) | 1.47 (0.73-2.93) | 1.37 (0.69-2.73) | 1.50 (0.77-2.91) | 1.30 (0.68-2.45) | 1.57 (0.82-3.00) | 1.77 (0.84-3.73) |
| Bipolar | 1.47 (0.81-2.69) | 1.33 (0.76-2.34) | 1.55 (0.93-2.58) | 1.56 (0.96-2.51) | 1.68 (1.06-2.66) | 2.08 (1.27-3.39) | 2.14 (1.23-3.72) |
| Other psychoses | 1.28 (0.73-2.24) | 1.13 (0.65-1.96) | 1.29 (0.77-2.17) | 1.51 (0.94-2.41) | 1.94 (1.26-3.00) | 1.97 (1.25-3.11) | 1.83 (1.09-3.07) |
| **Parkinsonism (other)** | |  |  |  |  |  |  |
| **All SMI** | 7.63 (3.63-16.02) | 9.59 (5.23-17.61) | 6.25 (3.96-9.85) | 8.42 (5.70-12.43) | 10.67 (7.69-14.80) | 13.66 (9.58-19.48) | 16.78 (11.13-25.30) |
| **Movement disorders (other)** | | | | | | | |
| **All SMI** | 1.92 (1.19-3.10) | 2.34 (1.52-3.61) | 2.49 (1.68-3.70) | 3.03 (2.09-4.37) | 3.69 (2.57-5.29) | 3.95 (2.67-5.84) | 4.19 (2.79-6.30) |
| **Motor Neuron Disease** | |  |  |  |  |  |  |
| **All SMI** | 0.71 (0.21-2.41) | 0.52 (0.16-1.74) | 0.71 (0.28-1.85) | 0.71 (0.28-1.85) | 1.55 (0.58-4.13) | 1.39 (0.45-4.32) | 1.69 (0.65-4.39) |
| **Autonomic nervous system disorders** | | | | | | | |
| **All SMI** | 1.60 (0.70-3.67) | 1.90 (0.89-4.09) | 1.82 (0.85-3.88) | 2.00 (0.96-4.17) | 2.00 (0.96-4.16) | 3.45 (1.52-7.82) | 3.50 (1.35-9.07) |

### Supplementary Table 6: Relative prevalence of neurological conditions in people with SMI and SMI subtypes to the comparator population (odds ratios adjusted for sex, age at SMI diagnosis, ethnicity, region and year of SMI diagnosis)

|  | **Years before SMI diagnosis** | |  | **Index date** | **Years after SMI diagnosis** | | |
| --- | --- | --- | --- | --- | --- | --- | --- |
|  | **-5** | **-3** | **-1** |  | **1** | **3** | **5** |
| **Multiple Sclerosis** | |  |  |  |  |  |  |
| **All SMI** | **1.36 (1.13-1.62)** | **1.36 (1.15-1.61)** | **1.41 (1.21-1.65)** | **1.43 (1.23-1.66)** | **1.57 (1.35-1.82)** | **1.52 (1.30-1.79)** | **1.43 (1.19-1.72)** |
| Schizophrenia | 0.62 (0.33-1.17) | 0.71 (0.40-1.24) | 0.71 (0.43-1.19) | 0.86 (0.54-1.38) | 1.03 (0.67-1.57) | 0.83 (0.51-1.35) | 0.72 (0.40-1.29) |
| Bipolar | 1.34 (1.01-1.76) | 1.38 (1.06-1.79) | 1.45 (1.14-1.85) | 1.44 (1.13-1.82) | 1.65 (1.31-2.08) | 1.68 (1.33-2.13) | 1.62 (1.23-2.13) |
| Other psychoses | 1.65 (1.27-2.14) | 1.56 (1.22-2.01) | 1.64 (1.30-2.07) | 1.63 (1.30-2.04) | 1.68 (1.34-2.10) | 1.78 (1.40-2.26) | 1.47 (1.10-1.97) |
| **Cerebrovascular disease** | | | | | | | |
| **All SMI** | **1.14 (1.06-1.23)** | **1.16 (1.09-1.24)** | **1.22 (1.16-1.29)** | **1.35 (1.28-1.42)** | **1.45 (1.38-1.52)** | **1.59 (1.51-1.67)** | **1.63 (1.53-1.73)** |
| Schizophrenia | 0.89 (0.73-1.09) | 0.93 (0.78-1.11) | 0.94 (0.81-1.11) | 1.00 (0.87-1.16) | 1.20 (1.05-1.37) | 1.29 (1.13-1.49) | 1.42 (1.22-1.65) |
| Bipolar | 1.08 (0.94-1.25) | 1.14 (1.01-1.29) | 1.34 (1.21-1.49) | 1.48 (1.35-1.63) | 1.64 (1.50-1.79) | 1.83 (1.68-2.00) | 1.64 (1.49-1.80) |
| Other psychoses | 1.23 (1.12-1.35) | 1.23 (1.13-1.33) | 1.25 (1.16-1.34) | 1.38 (1.29-1.48) | 1.44 (1.35-1.53) | 1.47 (1.37-1.57) | 1.30 (1.20-1.42) |
| **Dementia** |  |  |  |  |  |  |  |
| **All SMI** | **1.12 (0.88-1.42)** | **1.27 (1.07-1.51)** | **1.72 (1.55-1.91)** | **2.54 (2.34-2.76)** | **4.10 (3.84-4.37)** | **4.85 (4.53-5.19)** | **4.22 (3.88-4.58)** |
| Schizophrenia | 1.87 (1.06-3.30) | 1.60 (1.06-2.43) | 1.61 (1.20-2.16) | 2.30 (1.84-2.87) | 3.62 (3.03-4.33) | 4.32 (3.60-5.17) | 3.65 (2.96-4.51) |
| Bipolar | 1.90 (1.17-3.10) | 1.84 (1.22-2.77) | 2.08 (1.58-2.73) | 2.79 (2.29-3.40) | 3.87 (3.31-4.53) | 4.08 (3.51-4.74) | 3.22 (2.73-3.79) |
| Other psychoses | 0.84 (0.63-1.11) | 1.09 (0.90-1.31) | 1.66 (1.48-1.87) | 2.55 (2.32-2.81) | 4.38 (4.06-4.73) | 4.78 (4.40-5.19) | 3.53 (3.19-3.90) |
| **Ataxia** |  |  |  |  |  |  |  |
| **All SMI** | **2.12 (1.58-2.85)** | **1.94 (1.48-2.56)** | **1.81 (1.40-2.34)** | **1.91 (1.50-2.43)** | **2.27 (1.79-2.89)** | **2.73 (2.12-3.50)** | **2.72 (2.08-3.55)** |
| Schizophrenia | 1.81 (0.84-3.88) | 1.80 (0.92-3.52) | 1.63 (0.87-3.07) | 1.66 (0.90-3.06) | 1.86 (1.08-3.19) | 1.65 (0.93-2.92) | 1.61 (0.84-3.09) |
| Bipolar | 1.63 (0.90-2.96) | 1.72 (1.02-2.93) | 1.62 (0.97-2.71) | 1.71 (1.04-2.80) | 2.20 (1.40-3.46) | 2.59 (1.67-4.03) | 2.80 (1.73-4.51) |
| Other psychoses | 2.54 (1.72-3.76) | 2.13 (1.49-3.07) | 1.99 (1.42-2.78) | 2.11 (1.53-2.90) | 2.48 (1.81-3.39) | 3.38 (2.44-4.67) | 2.98 (2.05-4.33) |
| **Epilepsy** |  |  |  |  |  |  |  |
| **All SMI** | **2.15 (2.03-2.28)** | **2.21 (2.10-2.34)** | **2.27 (2.16-2.40)** | **2.40 (2.28-2.52)** | **2.70 (2.57-2.83)** | **2.87 (2.73-3.03)** | **3.01 (2.83-3.19)** |
| Schizophrenia | 2.09 (1.83-2.39) | 2.19 (1.93-2.50) | 2.20 (1.94-2.48) | 2.27 (2.02-2.55) | 2.76 (2.48-3.08) | 2.88 (2.57-3.21) | 3.13 (2.76-3.55) |
| Bipolar | 1.74 (1.56-1.93) | 1.81 (1.64-2.00) | 1.98 (1.80-2.17) | 2.13 (1.95-2.33) | 2.40 (2.20-2.61) | 2.75 (2.51-3.01) | 2.73 (2.46-3.03) |
| Other psychoses | 2.53 (2.33-2.74) | 2.56 (2.37-2.76) | 2.55 (2.37-2.74) | 2.68 (2.50-2.87) | 2.92 (2.73-3.12) | 3.23 (3.00-3.48) | 3.13 (2.87-3.42) |
|  | | | | | | | |
|  | | | | | | | |
| **Parkinson's disease** | | | | | | | |
| **All SMI** | **3.09 (2.60-3.67)** | **3.10 (2.66-3.62)** | **2.81 (2.45-3.21)** | **2.96 (2.62-3.34)** | **3.25 (2.91-3.63)** | **3.75 (3.35-4.20)** | **3.97 (3.45-4.57)** |
| Schizophrenia | 1.66 (1.03-2.69) | 1.54 (1.00-2.37) | 1.53 (1.03-2.27) | 2.02 (1.45-2.82) | 2.66 (2.01-3.52) | 4.12 (3.08-5.50) | 4.25 (3.05-5.92) |
| Bipolar | 1.33 (0.88-2.01) | 1.43 (1.00-2.03) | 1.69 (1.26-2.25) | 1.84 (1.41-2.40) | 2.29 (1.82-2.89) | 2.75 (2.16-3.50) | 2.71 (2.06-3.56) |
| Other psychoses | 4.55 (3.65-5.67) | 4.56 (3.74-5.57) | 3.70 (3.12-4.38) | 3.77 (3.23-4.40) | 3.91 (3.39-4.51) | 4.03 (3.48-4.68) | 3.83 (3.14-4.66) |
| **Paralysis** |  |  |  |  |  |  |  |
| **All SMI** | **1.78 (1.48-2.15)** | **1.77 (1.49-2.11)** | **1.75 (1.48-2.06)** | **1.79 (1.52-2.10)** | **1.88 (1.60-2.20)** | **1.99 (1.68-2.35)** | **1.76 (1.45-2.13)** |
| Schizophrenia | 1.20 (0.73-1.98) | 1.07 (0.66-1.73) | 1.13 (0.72-1.78) | 1.16 (0.75-1.79) | 1.23 (0.81-1.87) | 1.13 (0.73-1.74) | 1.14 (0.70-1.84) |
| Bipolar | 1.73 (1.27-2.35) | 1.79 (1.34-2.39) | 1.69 (1.28-2.23) | 1.74 (1.34-2.28) | 1.89 (1.46-2.45) | 2.15 (1.65-2.82) | 1.80 (1.32-2.45) |
| Other psychoses | 2.01 (1.57-2.56) | 2.01 (1.59-2.55) | 2.01 (1.60-2.52) | 2.04 (1.64-2.55) | 2.10 (1.69-2.61) | 2.37 (1.88-2.99) | 1.94 (1.47-2.57) |
| **Cerebral palsy** |  |  |  |  |  |  |  |
| **All SMI** | **2.56 (2.09-3.12)** | **2.50 (2.05-3.04)** | **2.48 (2.04-3.02)** | **2.52 (2.08-3.05)** | **2.64 (2.17-3.20)** | **2.43 (1.97-2.99)** | **2.34 (1.83-3.00)** |
| Schizophrenia | 1.85 (1.15-2.99) | 1.88 (1.18-2.98) | 1.88 (1.19-2.96) | 1.95 (1.25-3.03) | 1.95 (1.25-3.03) | 1.72 (1.07-2.76) | 1.48 (0.82-2.65) |
| Bipolar | 2.72 (1.97-3.76) | 2.66 (1.93-3.67) | 2.58 (1.87-3.55) | 2.55 (1.86-3.52) | 2.78 (2.02-3.81) | 2.91 (2.04-4.13) | 2.32 (1.56-3.47) |
| Other psychoses | 2.76 (2.04-3.74) | 2.68 (1.99-3.63) | 2.71 (2.01-3.65) | 2.81 (2.10-3.76) | 2.90 (2.17-3.88) | 2.85 (2.08-3.90) | 3.28 (2.21-4.84) |
| **CSF disorders** |  |  |  |  |  |  |  |
| **All SMI** | **1.80 (1.31-2.46)** | **1.75 (1.29-2.38)** | **1.81 (1.35-2.44)** | **1.96 (1.47-2.62)** | **2.21 (1.64-2.99)** | **2.26 (1.63-3.13)** | **2.34 (1.65-3.30)** |
| Schizophrenia | 1.56 (0.81-2.99) | 1.48 (0.80-2.75) | 1.51 (0.83-2.74) | 1.69 (0.95-2.99) | 1.94 (1.13-3.33) | 2.00 (1.16-3.47) | 2.13 (1.12-4.05) |
| Bipolar | 1.99 (1.18-3.36) | 1.87 (1.13-3.10) | 1.82 (1.12-2.97) | 1.90 (1.17-3.08) | 1.95 (1.21-3.14) | 2.09 (1.28-3.44) | 2.22 (1.28-3.85) |
| Other psychoses | 1.81 (1.10-2.99) | 1.84 (1.12-3.01) | 2.05 (1.31-3.22) | 2.24 (1.45-3.46) | 2.67 (1.75-4.08) | 2.91 (1.80-4.72) | 2.69 (1.59-4.54) |
| **Spinal cord disorders** | | | | | | | |
| **All SMI** | **1.30 (0.84-2.02)** | **1.37 (0.92-2.04)** | **1.44 (1.01-2.04)** | **1.59 (1.15-2.20)** | **1.70 (1.25-2.31)** | **1.70 (1.22-2.38)** | **1.45 (0.99-2.12)** |
| Schizophrenia | 0.85 (0.24-3.00) | 0.71 (0.21-2.44) | 0.89 (0.32-2.45) | 1.08 (0.42-2.81) | 1.39 (0.63-3.10) | 1.15 (0.47-2.82) | 1.63 (0.65-4.12) |
| Bipolar | 2.17 (1.08-4.33) | 2.47 (1.33-4.59) | 2.09 (1.19-3.65) | 2.14 (1.25-3.65) | 2.15 (1.29-3.57) | 2.30 (1.36-3.89) | 1.71 (0.97-3.01) |
| Other psychoses | 0.91 (0.44-1.88) | 0.94 (0.49-1.80) | 1.17 (0.68-2.01) | 1.36 (0.85-2.20) | 1.44 (0.92-2.25) | 1.52 (0.94-2.43) | 1.09 (0.57-2.06) |
|  | | | | | | | |
|  | | | | | | | |
|  | | | | | | | |
| **Disorders of nerve root, nerve plexus and peripheral nervous system** | | | | | | | |
| **All SMI** | **1.38 (0.96-1.98)** | **1.26 (0.90-1.78)** | **1.38 (1.00-1.92)** | **1.51 (1.11-2.05)** | **1.70 (1.26-2.30)** | **1.96 (1.44-2.68)** | **2.03 (1.46-2.83)** |
| Schizophrenia | 1.47 (0.70-3.08) | 1.45 (0.71-2.94) | 1.35 (0.67-2.72) | 1.47 (0.74-2.90) | 1.25 (0.65-2.42) | 1.58 (0.82-3.06) | 1.86 (0.87-3.99) |
| Bipolar | 1.49 (0.82-2.72) | 1.34 (0.76-2.35) | 1.55 (0.93-2.58) | 1.55 (0.96-2.50) | 1.70 (1.07-2.68) | 2.27 (1.39-3.72) | 2.24 (1.29-3.90) |
| Other psychoses | 1.26 (0.72-2.20) | 1.11 (0.64-1.93) | 1.26 (0.75-2.13) | 1.48 (0.92-2.37) | 1.92 (1.24-2.97) | 2.04 (1.29-3.23) | 1.79 (1.07-3.01) |
| **Parkinsonism (other)** | | | | | | | |
| **All SMI** | **7.70 (3.68-16.13)** | **9.71 (5.31-17.78)** | **6.25 (3.97-9.85)** | **8.32 (5.64-12.29)** | **10.59 (7.63-14.70)** | **14.51 (10.16-20.72)** | **19.08 (12.65-28.78)** |
| **Movement disorders (other)** | | | | | | | |
| **All SMI** | **1.93 (1.20-3.12)** | **2.35 (1.53-3.62)** | **2.50 (1.68-3.71)** | **3.03 (2.10-4.39)** | **3.71 (2.59-5.33)** | **4.13 (2.78-6.14)** | **4.42 (2.94-6.63)** |
| **Motor Neuron Disease** | | | | | | | |
| **All SMI** | **0.72 (0.21-2.51)** | **0.53 (0.16-1.78)** | **0.72 (0.28-1.90)** | **0.72 (0.28-1.90)** | **1.59 (0.58-4.35)** | **1.50 (0.47-4.78)** | **1.79 (0.68-4.69)** |
| **Autonomic nervous system disorders** | | | | | | | |
| **All SMI** | **1.58 (0.68-3.66)** | **1.89 (0.87-4.09)** | **1.80 (0.84-3.87)** | **1.98 (0.94-4.16)** | **1.98 (0.94-4.16)** | **3.40 (1.51-7.66)** | **3.39 (1.32-8.70)** |

### Supplementary Table 7: Relative prevalence of neurological conditions in people with SMI and SMI subtypes compared to the comparator population (odds ratios adjusted for sex, age at SMI diagnosis, ethnicity, region, year of SMI diagnosis, BMI, smoking status, alcohol misuse and substance misuse)

|  | **Years before SMI diagnosis** | |  | **Index date** | **Years after SMI diagnosis** | | |
| --- | --- | --- | --- | --- | --- | --- | --- |
|  | -5 | -3 | -1 |  | 1 | 3 | 5 |
| **Multiple Sclerosis** |  |  |  |  |  |  |  |
| **All SMI** | 1.36 (1.13-1.63) | 1.36 (1.14-1.61) | 1.41 (1.20-1.66) | 1.43 (1.23-1.66) | 1.52 (1.32-1.76) | 1.45 (1.24-1.70) | 1.41 (1.17-1.70) |
| Schizophrenia | 0.63 (0.34-1.16) | 0.73 (0.42-1.27) | 0.75 (0.45-1.24) | 0.90 (0.57-1.43) | 1.07 (0.70-1.63) | 0.85 (0.53-1.36) | 0.76 (0.43-1.36) |
| Bipolar | 1.31 (0.99-1.74) | 1.35 (1.03-1.76) | 1.42 (1.11-1.82) | 1.40 (1.10-1.79) | 1.58 (1.25-2.00) | 1.59 (1.26-2.02) | 1.62 (1.23-2.13) |
| Other psychoses | 1.68 (1.28-2.19) | 1.57 (1.22-2.03) | 1.65 (1.30-2.09) | 1.64 (1.30-2.06) | 1.63 (1.30-2.03) | 1.68 (1.32-2.14) | 1.41 (1.05-1.89) |
| **Cerebrovascular disease** | |  |  |  |  |  |  |
| **All SMI** | 1.12 (1.04-1.22) | 1.14 (1.06-1.22) | 1.20 (1.13-1.27) | 1.32 (1.25-1.39) | 1.42 (1.35-1.49) | 1.56 (1.48-1.64) | 1.58 (1.48-1.68) |
| Schizophrenia | 0.83 (0.67-1.02) | 0.87 (0.72-1.05) | 0.88 (0.75-1.04) | 0.94 (0.81-1.10) | 1.14 (0.99-1.31) | 1.23 (1.06-1.43) | 1.38 (1.18-1.61) |
| Bipolar | 1.05 (0.90-1.22) | 1.09 (0.96-1.24) | 1.28 (1.15-1.43) | 1.42 (1.28-1.57) | 1.57 (1.43-1.72) | 1.79 (1.63-1.96) | 1.58 (1.43-1.75) |
| Other psychoses | 1.24 (1.12-1.37) | 1.23 (1.13-1.34) | 1.25 (1.15-1.35) | 1.38 (1.28-1.48) | 1.43 (1.33-1.52) | 1.46 (1.36-1.57) | 1.28 (1.18-1.40) |
| **Dementia** |  |  |  |  |  |  |  |
| **All SMI** | 1.18 (0.93-1.51) | 1.37 (1.15-1.64) | 1.86 (1.66-2.07) | 2.78 (2.54-3.03) | 4.54 (4.24-4.86) | 5.32 (4.96-5.72) | 4.50 (4.13-4.91) |
| Schizophrenia | 1.94 (1.11-3.38) | 1.77 (1.16-2.70) | 1.79 (1.32-2.42) | 2.63 (2.09-3.31) | 4.19 (3.47-5.05) | 4.83 (4.00-5.83) | 3.93 (3.15-4.90) |
| Bipolar | 2.09 (1.22-3.57) | 2.06 (1.31-3.24) | 2.30 (1.70-3.11) | 3.08 (2.49-3.81) | 4.28 (3.63-5.04) | 4.45 (3.81-5.20) | 3.41 (2.88-4.04) |
| Other psychoses | 0.85 (0.64-1.14) | 1.12 (0.92-1.36) | 1.72 (1.52-1.95) | 2.69 (2.42-2.98) | 4.71 (4.35-5.10) | 5.10 (4.68-5.55) | 3.69 (3.33-4.10) |
| **Ataxia** |  |  |  |  |  |  |  |
| **All SMI** | 1.90 (1.35-2.67) | 1.69 (1.22-2.34) | 1.47 (1.09-1.99) | 1.56 (1.17-2.07) | 1.80 (1.39-2.33) | 2.24 (1.72-2.91) | 2.34 (1.74-3.14) |
| Schizophrenia | 1.56 (0.70-3.49) | 1.64 (0.81-3.33) | 1.42 (0.73-2.76) | 1.44 (0.75-2.78) | 1.66 (0.94-2.93) | 1.57 (0.88-2.81) | 1.54 (0.80-2.95) |
| Bipolar | 1.52 (0.75-3.07) | 1.50 (0.76-2.94) | 1.34 (0.70-2.56) | 1.43 (0.77-2.66) | 1.77 (1.02-3.05) | 2.23 (1.37-3.64) | 2.65 (1.55-4.51) |
| Other psychoses | 2.26 (1.45-3.53) | 1.84 (1.20-2.82) | 1.57 (1.06-2.34) | 1.68 (1.15-2.45) | 1.90 (1.34-2.68) | 2.66 (1.87-3.79) | 2.44 (1.62-3.69) |
| **Epilepsy** |  |  |  |  |  |  |  |
| **All SMI** | 2.02 (1.90-2.14) | 2.05 (1.94-2.18) | 2.06 (1.95-2.18) | 2.16 (2.05-2.28) | 2.44 (2.32-2.57) | 2.63 (2.49-2.78) | 2.77 (2.60-2.96) |
| Schizophrenia | 1.95 (1.69-2.25) | 2.01 (1.75-2.32) | 1.96 (1.71-2.24) | 2.02 (1.78-2.30) | 2.51 (2.23-2.82) | 2.66 (2.37-3.00) | 2.94 (2.57-3.37) |
| Bipolar | 1.63 (1.46-1.82) | 1.68 (1.51-1.87) | 1.80 (1.63-1.99) | 1.93 (1.75-2.12) | 2.18 (1.99-2.39) | 2.52 (2.29-2.78) | 2.52 (2.26-2.81) |
| Other psychoses | 2.40 (2.20-2.61) | 2.40 (2.21-2.60) | 2.36 (2.18-2.55) | 2.44 (2.26-2.63) | 2.66 (2.47-2.85) | 2.99 (2.76-3.23) | 2.94 (2.68-3.23) |
| **Parkinson's disease** |  |  |  |  |  |  |  |
| **All SMI** | 3.47 (2.90-4.15) | 3.47 (2.96-4.07) | 3.10 (2.69-3.56) | 3.24 (2.86-3.69) | 3.54 (3.16-3.97) | 4.05 (3.59-4.55) | 4.26 (3.67-4.93) |
| Schizophrenia | 1.75 (1.08-2.86) | 1.60 (1.02-2.52) | 1.63 (1.08-2.45) | 2.17 (1.54-3.05) | 2.91 (2.18-3.89) | 4.52 (3.36-6.08) | 4.71 (3.37-6.59) |
| Bipolar | 1.43 (0.94-2.19) | 1.52 (1.05-2.18) | 1.79 (1.33-2.41) | 1.93 (1.47-2.55) | 2.40 (1.90-3.05) | 2.85 (2.23-3.66) | 2.83 (2.14-3.74) |
| Other psychoses | 5.15 (4.10-6.46) | 5.18 (4.22-6.36) | 4.12 (3.46-4.91) | 4.17 (3.55-4.90) | 4.29 (3.70-4.98) | 4.38 (3.75-5.12) | 4.14 (3.37-5.08) |
|  |  |  |  |  |  |  |  |
|  |  |  |  |  |  |  |  |
|  | **Years before SMI diagnosis** | |  | **Index date** | **Years after SMI diagnosis** | | |
|  | -5 | -3 | -1 |  | 1 | 3 | 5 |
| **Paralysis** |  |  |  |  |  |  |  |
| **All SMI** | 1.84 (1.51-2.25) | 1.82 (1.51-2.20) | 1.78 (1.49-2.13) | 1.83 (1.54-2.18) | 1.87 (1.58-2.21) | 1.95 (1.64-2.32) | 1.77 (1.43-2.17) |
| Schizophrenia | 1.40 (0.83-2.37) | 1.20 (0.72-2.00) | 1.27 (0.78-2.06) | 1.30 (0.82-2.07) | 1.36 (0.87-2.11) | 1.27 (0.81-2.01) | 1.28 (0.77-2.13) |
| Bipolar | 1.77 (1.27-2.46) | 1.83 (1.34-2.49) | 1.69 (1.26-2.28) | 1.76 (1.33-2.34) | 1.87 (1.42-2.45) | 2.08 (1.58-2.74) | 1.77 (1.27-2.47) |
| Other psychoses | 2.04 (1.57-2.66) | 2.03 (1.58-2.62) | 2.03 (1.59-2.59) | 2.08 (1.64-2.63) | 2.07 (1.64-2.61) | 2.28 (1.78-2.93) | 1.92 (1.42-2.60) |
| **Cerebral palsy** |  |  |  |  |  |  |  |
| **All SMI** | 2.88 (2.34-3.54) | 2.82 (2.30-3.46) | 2.79 (2.27-3.42) | 2.85 (2.33-3.48) | 2.92 (2.39-3.56) | 2.66 (2.15-3.28) | 2.68 (2.08-3.46) |
| Schizophrenia | 2.02 (1.23-3.31) | 2.07 (1.28-3.35) | 2.08 (1.30-3.33) | 2.16 (1.36-3.42) | 2.16 (1.36-3.42) | 1.92 (1.18-3.12) | 1.62 (0.89-2.95) |
| Bipolar | 3.14 (2.24-4.41) | 3.07 (2.19-4.31) | 2.98 (2.13-4.17) | 2.96 (2.12-4.14) | 3.14 (2.26-4.37) | 3.23 (2.25-4.65) | 2.77 (1.83-4.19) |
| Other psychoses | 3.13 (2.26-4.33) | 3.05 (2.21-4.20) | 3.04 (2.21-4.19) | 3.18 (2.33-4.34) | 3.20 (2.35-4.36) | 3.09 (2.22-4.30) | 3.72 (2.49-5.55) |
| **CSF disorders** |  |  |  |  |  |  |  |
| **All SMI** | 1.89 (1.34-2.67) | 1.82 (1.31-2.54) | 1.89 (1.37-2.61) | 2.05 (1.50-2.81) | 2.21 (1.66-2.95) | 2.18 (1.59-2.99) | 2.43 (1.66-3.54) |
| Schizophrenia | 1.53 (0.75-3.10) | 1.44 (0.73-2.81) | 1.50 (0.79-2.87) | 1.70 (0.92-3.17) | 1.98 (1.11-3.52) | 2.05 (1.14-3.69) | 2.53 (1.30-4.91) |
| Bipolar | 2.26 (1.29-3.97) | 2.09 (1.22-3.60) | 2.04 (1.21-3.45) | 2.14 (1.27-3.58) | 2.00 (1.24-3.22) | 2.01 (1.21-3.32) | 2.28 (1.23-4.21) |
| Other psychoses | 1.88 (1.10-3.23) | 1.88 (1.11-3.20) | 2.06 (1.26-3.38) | 2.27 (1.42-3.62) | 2.62 (1.71-4.02) | 2.78 (1.71-4.52) | 2.67 (1.51-4.71) |
| **Spinal cord disorders** | |  |  |  |  |  |  |
| **All SMI** | 1.37 (0.86-2.17) | 1.44 (0.95-2.19) | 1.46 (1.01-2.10) | 1.64 (1.17-2.30) | 1.65 (1.22-2.23) | 1.60 (1.15-2.22) | 1.46 (0.98-2.18) |
| Schizophrenia | 0.82 (0.24-2.83) | 0.71 (0.21-2.39) | 0.86 (0.32-2.31) | 1.06 (0.42-2.71) | 1.38 (0.62-3.03) | 1.14 (0.48-2.75) | 1.50 (0.60-3.73) |
| Bipolar | 2.34 (1.15-4.77) | 2.70 (1.43-5.08) | 2.20 (1.24-3.89) | 2.26 (1.31-3.90) | 2.02 (1.23-3.32) | 2.12 (1.27-3.53) | 1.87 (1.04-3.34) |
| Other psychoses | 0.96 (0.43-2.13) | 0.98 (0.48-1.99) | 1.18 (0.66-2.14) | 1.42 (0.85-2.38) | 1.46 (0.90-2.37) | 1.47 (0.87-2.47) | 1.05 (0.53-2.07) |
| **Disorders of nerve root, nerve plexus and peripheral nervous system** | | | | | | | |
| **All SMI** | 1.13 (0.79-1.62) | 1.01 (0.72-1.43) | 1.08 (0.77-1.50) | 1.16 (0.85-1.58) | 1.25 (0.94-1.65) | 1.51 (1.13-2.03) | 1.74 (1.23-2.45) |
| Schizophrenia | 1.33 (0.63-2.79) | 1.29 (0.63-2.64) | 1.23 (0.61-2.48) | 1.36 (0.68-2.72) | 1.09 (0.56-2.12) | 1.43 (0.74-2.78) | 1.61 (0.76-3.44) |
| Bipolar | 1.20 (0.66-2.19) | 1.04 (0.59-1.83) | 1.20 (0.70-2.04) | 1.18 (0.72-1.95) | 1.24 (0.77-2.00) | 1.69 (1.01-2.83) | 1.93 (1.06-3.49) |
| Other psychoses | 1.00 (0.56-1.78) | 0.87 (0.49-1.53) | 0.93 (0.54-1.61) | 1.06 (0.65-1.74) | 1.35 (0.87-2.08) | 1.56 (0.99-2.45) | 1.59 (0.94-2.67) |
| **Parkinsonism (other)** | |  |  |  |  |  |  |
| **All SMI** | 7.60 (3.62-15.94) | 9.91 (5.37-18.30) | 6.73 (4.24-10.67) | 8.90 (6.00-13.20) | 10.91 (7.88-15.12) | 14.99 (10.40-21.63) | 20.85 (13.71-31.71) |
| **Movement disorders (other)** | | | | | | | |
| **All SMI** | 1.91 (1.14-3.20) | 2.27 (1.42-3.62) | 2.49 (1.64-3.77) | 3.00 (2.03-4.42) | 3.45 (2.44-4.89) | 3.70 (2.52-5.42) | 4.34 (2.87-6.56) |
| **Motor Neuron Disease** | |  |  |  |  |  |  |
| **All SMI** | 0.82 (0.24-2.73) | 0.56 (0.16-1.91) | 0.79 (0.30-2.09) | 0.79 (0.30-2.09) | 1.16 (0.56-2.39) | 0.97 (0.43-2.17) | 1.98 (0.70-5.58) |
| **Autonomic nervous system disorders** | | | | | | | |
| **All SMI** | 1.65 (0.69-3.94) | 2.00 (0.90-4.43) | 1.92 (0.87-4.22) | 2.13 (0.99-4.58) | 2.13 (0.99-4.58) | 3.82 (1.69-8.64) | 3.90 (1.52-9.99) |

### Supplementary Figure 1: Directed acyclic graph


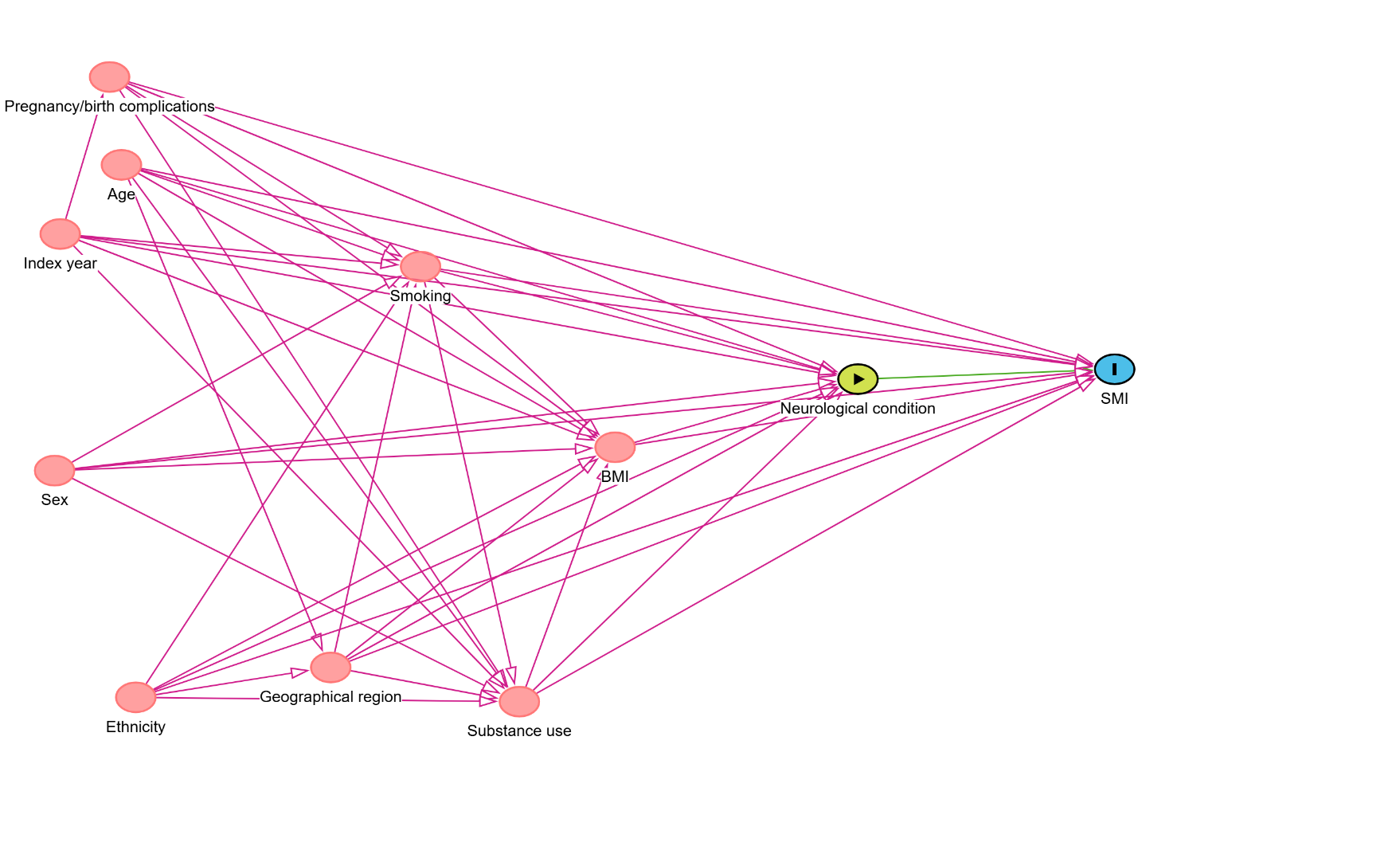


###
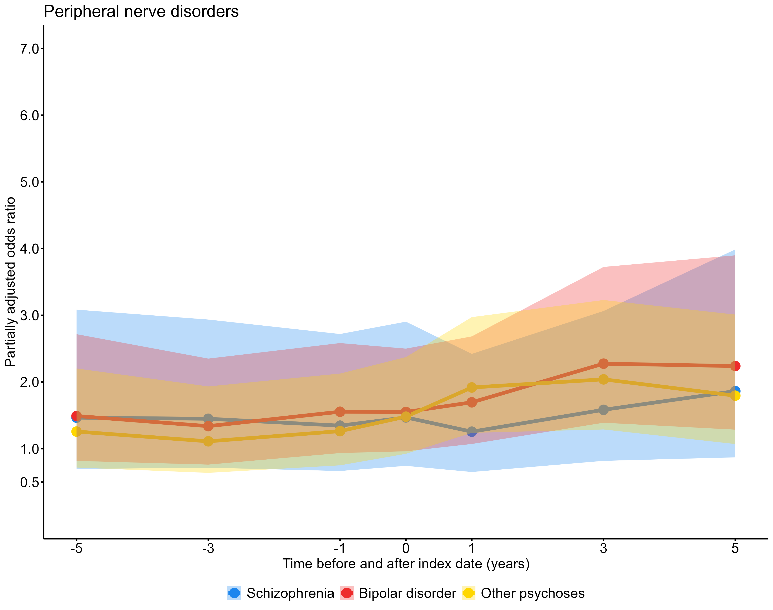
 Supplementary Figure 2: Odds ratios for other neurological conditions adjusted for sex, age at SMI diagnosis, ethnicity, region and year of SMI diagnosis at all time points for people with SMI compared to matched population without SMI


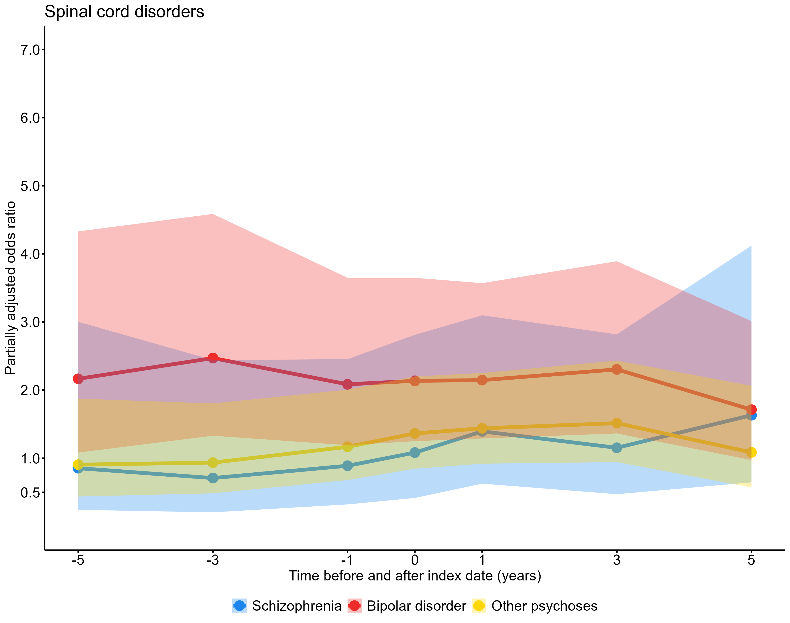

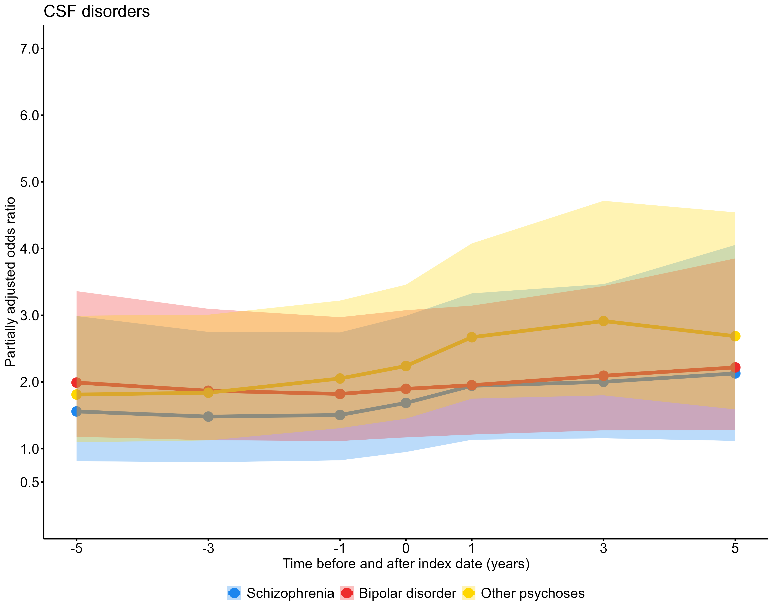

Supplement: Supplementary file 1 [file bmjment-28-1-s001.docx]
